# Spatio-temporal orientation of microtubules controls conical cell shape in *Arabidopsis thaliana* petals

**DOI:** 10.1371/journal.pgen.1006851

**Published:** 2017-06-23

**Authors:** Huibo Ren, Xie Dang, Xianzhi Cai, Peihang Yu, Yajun Li, Shanshan Zhang, Menghong Liu, Binqing Chen, Deshu Lin

**Affiliations:** 1 Basic Forestry and Proteomics Research Center, Fujian Provincial Key Laboratory of Haixia Applied Plant Systems Biology, College of Life Science, Fujian Agriculture and Forestry University, Fuzhou, China; 2 Haixia Institute of Science and Technology, Fujian Agriculture and Forestry University, Fuzhou, China; University of Florida, UNITED STATES

## Abstract

The physiological functions of epidermal cells are largely determined by their diverse morphologies. Most flowering plants have special conical-shaped petal epidermal cells that are thought to influence light capture and reflectance, and provide pollinator grips, but the molecular mechanisms controlling conical cell shape remain largely unknown. Here, we developed a live-confocal imaging approach to quantify geometric parameters of conical cells in *Arabidopsis thaliana* (*A*. *thaliana*). Through genetic screens, we identified *katanin* (*KTN1*) mutants showing a phenotype of decreased tip sharpening of conical cells. Furthermore, we demonstrated that SPIKE1 and Rho of Plants (ROP) GTPases were required for the final shape formation of conical cells, as KTN1 does. Live-cell imaging showed that wild-type cells exhibited random orientation of cortical microtubule arrays at early developmental stages but displayed a well-ordered circumferential orientation of microtubule arrays at later stages. By contrast, loss of KTN1 prevented random microtubule networks from shifting into well-ordered arrays. We further showed that the filamentous actin cap, which is a typical feature of several plant epidermal cell types including root hairs and leaf trichomes, was not observed in the growth apexes of conical cells during cell development. Moreover, our genetic and pharmacological data suggested that microtubules but not actin are required for conical cell shaping. Together, our results provide a novel imaging approach for studying petal conical cell morphogenesis and suggest that the spatio-temporal organization of microtubule arrays plays crucial roles in controlling conical cell shape.

## Author summary

How cells achieve their final shapes is a fundamental question in biology. Most flowering plants have special conical-shaped petal epidermal cells that are thought to attract pollinators, but the molecular and genetic mechanisms that control conical cell shape remain unknown. In this study, we developed a live-confocal imaging approach for the quantitative study of conical cell morphogenesis. Through genetic screens, we showed that *A*. *thaliana* KTN1, ROP GTPases, and SPIKE1 are required for conical cell shaping. Live-cell imaging showed that loss of KTN1 prevented random microtubule networks from shifting into well-ordered microtubule arrays at later developmental stages, which is correlated with the tip sharpening of conical cells. Moreover, genetic and pharmacological data suggested that microtubules but not actin are required for conical cell shaping. Together, our findings provide significant insights into the spatio-temporal organization of microtubules that controls conical cell development.

## Introduction

Plant epidermal cells have diverse shapes that enable these cells to perform unique physiological functions. Floral petals of nearly 79% of angiosperm species contain conical epidermal cells that are usually found on the adaxial epidermis (the upper surface), oriented towards potential pollinators but rarely present on leaves or any other organ epidermis [1–4]. Conical cells exhibit a three-dimensional (3D) geometric shape with a cone tip and a pentagonal or hexagonal base, which influences petal color, light capture and reflectance, petal wettability, and pollinator grips [5–8].However, despite the important physiological roles and the special shape of conical cells, little is known about the mechanisms that control their shape formation.

Currently, our knowledge of conical cells derived from images acquired by scanning electron microscopes or optical microscopes. The *MIXTA* gene encodes a MYB transcription factor in *Antirrhinum majus*, whose loss-of-function mutants result in petal epidermis with flat hexagonal-based cells instead of wild-type conical cells [9]. This change in cell morphology has been shown to reduce the mutant flowers’ chances of being visited by pollinators and thus affects pollination success [10]. *A*. *thaliana* studies using scanning electron microscopes to visualize epidermal cells have identified several transcriptional factors that function in regulating the outgrowth of conical cells [5, 11–16], but the molecular and genetic mechanisms controlling conical cell morphogenesis remain largely unknown.

Plant cells achieve their final shapes with the aid of cytoskeletal elements, which include actin filaments and microtubules [17]. Actin filaments play an important role in cell shape formation by guiding vesicle trafficking to promote cell elongation [18]. Cortical microtubules play a key role in orienting the deposition of cellulose microfibrils during cell wall biosynthesis and thus affect cell morphogenesis [19–22]. Owing to the advancement of live-cell imaging technologies, extensive studies have provided critical insights into the reorganization of microtubule arrays, an event that is in part mediated by self-organization processes involving severing, polymerization, depolymerization, and zippering [23–25]. The microtubule-severing protein KTN1 was originally identified from a screen for mutations that led to defects in the mechanical strength of inflorescence stems [26, 27]. Loss-of-function mutations of KTN1 result in a remarkable defect in leaf epidermal cell shape, associated with disordered microtubule arrays and abnormal orientation of cellulose microfibrils, as well as loss of layers in the secondary cell walls of fibers [26–28]. Previous results have shown that KTN1 is recruited to both the microtubule nucleation sites and microtubule crossovers to perform its microtubule-severing function, which is required for the generation of well-ordered microtubule arrays [29–31]. Moreover, it has been shown that KTN1 plays essential roles in organizing diverse patterns of microtubule arrays in response to mechanical stress [32, 33], and the environmental signal stimuli, such as blue light [34]. Despite the central roles of the cytoskeletal systems in regulating plant cell morphogenesis, functional analyses of cytoskeletal control of petal conical cell morphogenesis remain as a missing research field.

In contrast to the detailed understanding of molecular mechanisms that control the morphogenesis of diverse plant epidermal cell types [35–40], including leaf trichomes and pavement cells, and root hairs, the genetic and molecular mechanisms that control the morphogenesis of conical cells remain elusive, probably owing to the lack of available live-confocal scanning imaging approaches. In this study, we established a live-confocal scanning imaging approach for the quantitative study of conical cell morphogenesis. In addition, genetic and pharmacological experiments demonstrated that microtubules but not actin filaments play a major role in regulating formation of the final shape of conical cells. Our findings not only provide significant insights into the functional analysis of cytoskeletal control of the morphogenesis of flower petal conical cells, but also may pave the way for a new model system to study cell shape in *A*. *thaliana*.

## Results

### Developing an approach for quantification of conical cells' geometry by confocal laser scanning microscopy

**Fig 1:**
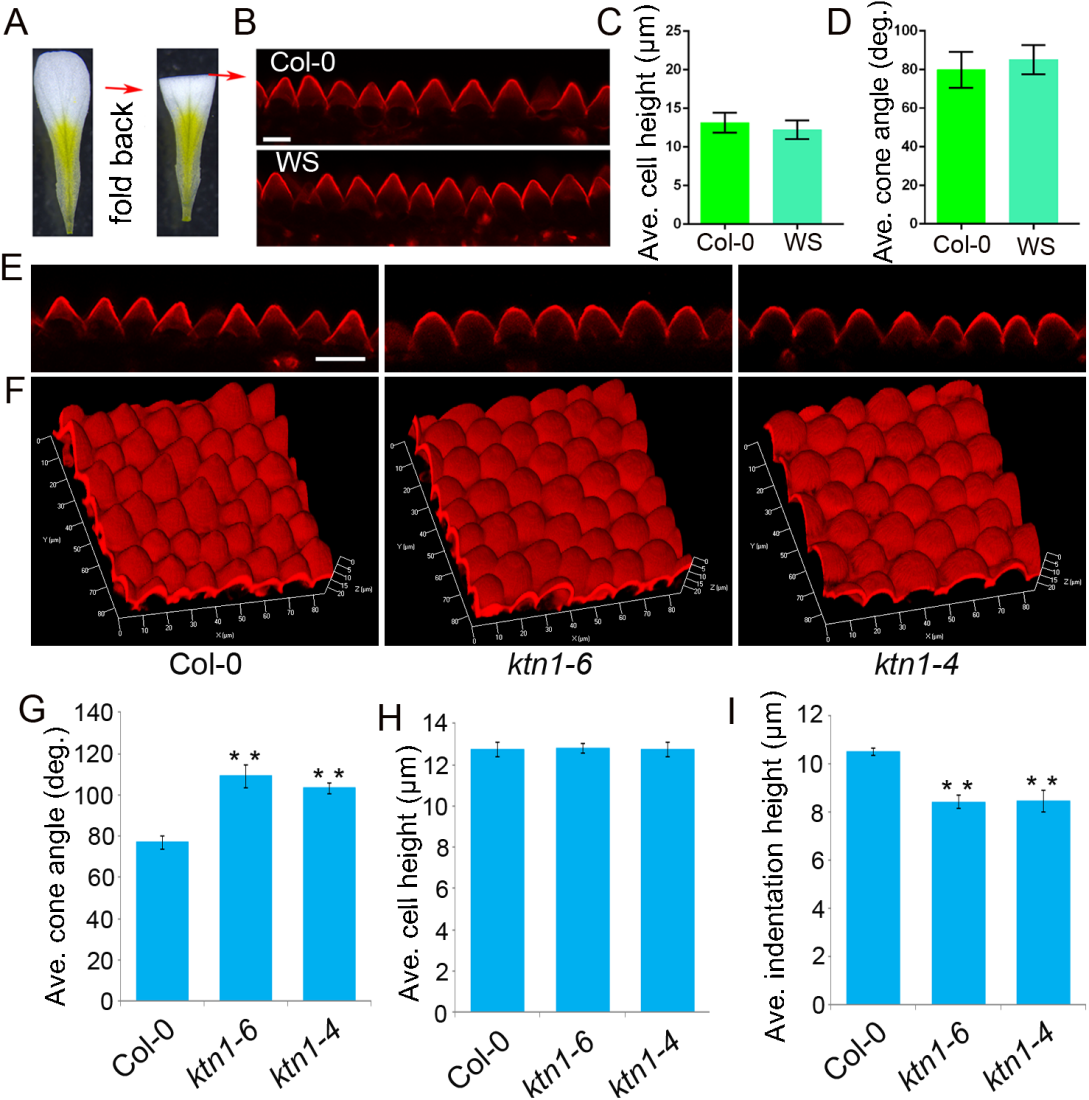
Developing an approach to quantify the geometry of conical cells with a confocal laser scanning microscope. (A) Schematic flowchart showing the folding back of a petal to expose the interface. *A*. *thaliana* petals are folded back to allow for the side view of conical cells at the folding position under a microscope. (B) Confocal images of conical cells from stage 14 petals of *A*. *thaliana* (Col-0 and WS). The side visualization of conical cells from the propidium iodide-stained folded petals by Zeiss LSM 880 confocal. Scale bar = 10μm. (C and D) Quantitative analyses of the geometry of conical cells. Cell heights (C) and cone angles (D) were quantified from confocal images. Values are given as the mean ± SD of more than 300 cells of 6 petals from independent plants. (E) Confocal images of petal conical cells of wild type, the *ktn1-6* mutant (the EMS mutant line), and the *ktn1-4* mutant (the T-DNA mutant line). Scale bar = 20μm. (F) 3D reconstruction of conical cells from wild type, *ktn1-6*, and *ktn1-4*. Z stacks of confocal images of PI-stained non-folded petals (stage 14) were taken from the top view along their Z axis at steps of 0.8 μm to reconstruct 3D images. (G–I) Quantitative analyses of conical cells from wild type, *ktn1-6*, and *ktn1-4*. Confocal images of conical cells from stage 14 petals of wild type and *ktn* mutants were used for quantification. Quantitative analysis showed that conical cells of the *ktn* mutants displayed a significant increase in cone angles (G) and decrease in indentation heights (I) compared with the wild type. Asterisk indicates a significant difference(student *t*-test, **P<0.01)between the data sets from *ktn1-6* and *ktn1-4* compared with Col-0 (P = 0.000472, P = 0.000736, respectively). Quantitative analysis of cell heights (H) showed that there were no significant differences between Col-0 and the *ktn* mutants (student *t*-test, P = 0.740, P = 0.920). Values are given as the mean ± SD of more than 300 cells of 6 petals from independent plants.

*A*. *thaliana* conical cells protrude outwards from the plane of the adaxial epidermis; therefore, the conical cells’ lateral cell walls that are not in the plane of the focal axis of the confocal laser scanning microscope cannot be observed from the top view of a conical cell from a petal sample that is faced up (S1A Fig), and only hexagonal outlines of the conical cell's basal part were visualized (S1B Fig). To make the lateral cell walls of conical cells into the focal plane of the microscope, petal blades were transversally folded back to expose the adaxial interface to the fold (Fig 1A and S1C Fig), which enables a side visualization of the conical cells (Fig 1B and S1D Fig). Z stacks of optical sections were taken from the top view of the adaxial epidermis from a folded petal, and projected onto a plane at maximum intensity to generate a quantifiable serrated shape of the conical cells (Fig 1B and S1D Fig). By contrast, exposing the abaxial interface to the fold resulted in the observation of flat abaxial epidermal cells (S1E Fig). These results are consistent with previous reports that the petal adaxial epidermis has conical cells while the abaxial epidermis has flat-shaped cells (S1F and S1G Fig) [3, 7].

We quantified the structural parameters of conical cells (S1H Fig), including cell heights and cone angles. We found that *A*. *thaliana* wild type Col-0 and WS conical cells from mature petals displayed similar cell heights, with 13μm on average, and the apex angles of these two ecotypes were 80°on average (Fig 1C and 1D).

To verify the accuracy and reproducibility of these analyses, we further quantified structural parameters using images of histological sections of fixed samples (S2 Fig). Our results showed that the cone structural parameters calculated using these two techniques were comparable to each other (S2 Fig). Therefore, we developed a live-confocal imaging approach for fast quantitative analyses of the structural parameters of conical cells.

### Loss of KTN1 causes swollen conical cell apexes

To uncover the genetic and molecular mechanisms controlling the shape formation of conical cells, we mutagenized wild-type Col-0 with ethyl methane sulfonate (EMS), and performed a genetic screen for mutants with abnormal conical cell shapes using our newly developed confocal-imaging approach. A mutant showing swollen apexes of conical cells compared with the wild type was identified (Fig 1E). We mapped the mutation to an interval of the short arm of chromosome 1, containing the *KTN1* locus. Sequencing of the *KTN1* gene itself revealed a C-to-T mutation, resulting in an A-to-V amino acid substitution (S3A and S3B Fig); this new mutation was designated as *ktn1-6*. To determine whether the phenotype of swollen conical cell apexes was caused by the mutation in *KTN1*, we performed a genetic complementation test. Expression of KTN1 by transforming *pKTN1*::*KTN1* into the *ktn1-6* mutant complemented its phenotype (S3C and S3D Fig). In addition, the T-DNA insertion mutant *ktn1-4* (SAIL_343_D12) for the *KTN1* gene (Lin et al., 2013) displayed the cell phenotype similar to the *ktn1-6* mutant (Fig 1E). Quantitative analyses revealed that both the *ktn1-6* and *ktn1-4* mutant showed increased radial expansion of conical cell apexes but no change in the basal parts in comparison with the wild type, (Fig 1G and S4A–S4E Fig), with larger cone angles and reduced heights in the gaps (indentation heights) between two neighboring cells (Fig 1G–1I). These observations suggest that KTN1 function is required for the morphogenesis of conical cells.

### KTN1 promotes the tip sharpening of conical cells during late development stages

The morphological events of petal development have been well characterized in *A*. *thaliana* [41], whereas the development of conical cells has not been described. We first characterized this process in wild-type *A*. *thaliana* petals via quantitative analyses of the serrated geometry of conical cells from various petal development stages. Because it is extremely difficult to track the same cell to observe changes in the cell morphology of growing petals, we measured temporal morphological changes of conical cells in the average height, width, and cone angle from petal development stage 8 to stage 14.

**Fig 2:**
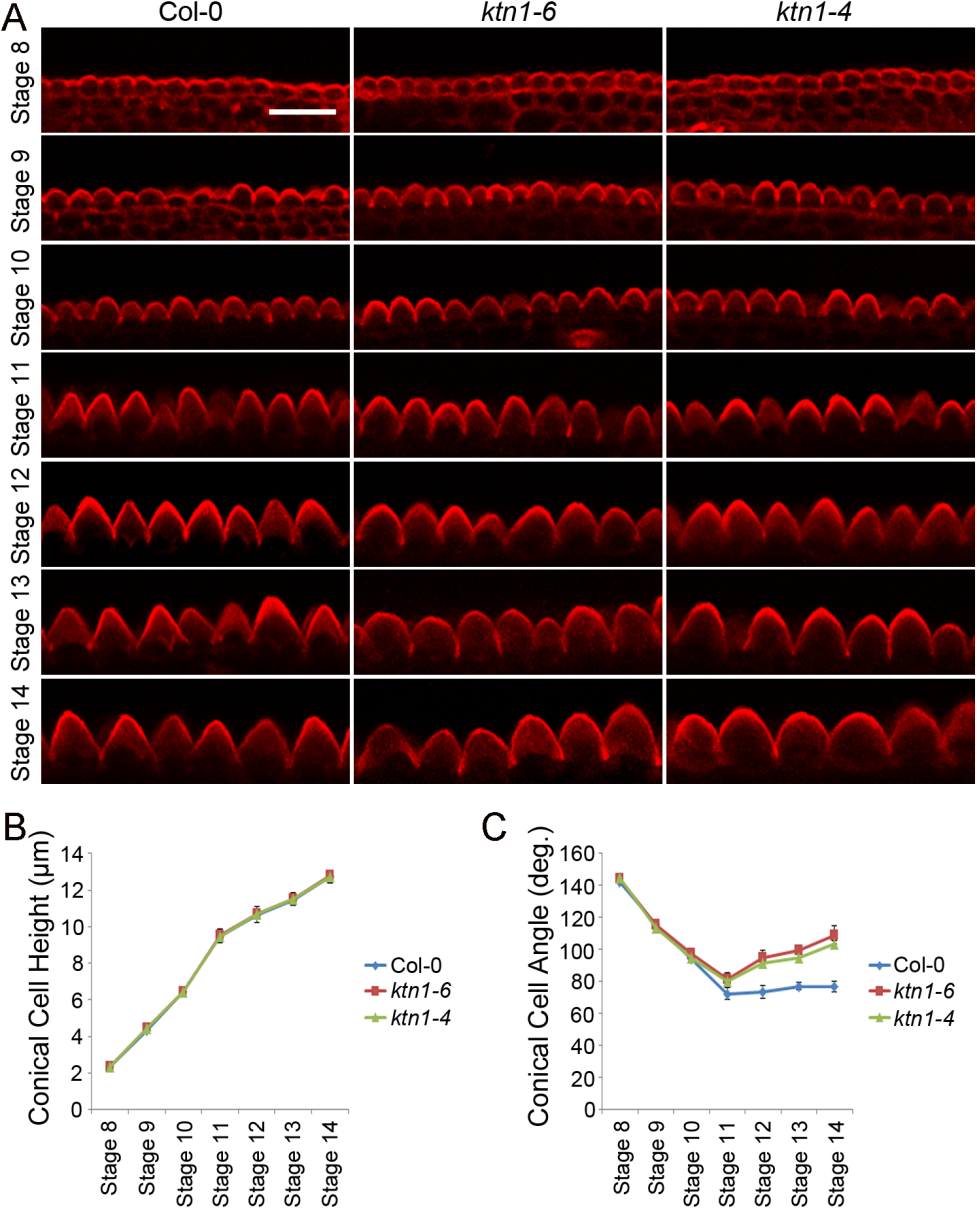
Comparison of conical cells' geometry of wild type and the *ktn* mutants at developmental stages 8–14. (A) Representative confocal images of conical cells at flower developmental stages 8–14 of wild type (Col-0), *ktn1-6*, and *ktn1-4*. Petals from wild type, *ktn1-6*, and *ktn1-4* flowers at stages 8 to 14 were carefully dissected, and petal blades were transversally folded back to expose the adaxial interface to the fold. Images of conical cells were captured from the side view via a confocal microscope. Scale bar = 20μm. (B and C) Quantitative analyses of conical cells from development stages 8 to 14 in wild type (Col-0), *ktn1-6*, and *ktn1-4*. Quantitative analysis of cell heights (B) by student *t*-test showed that there were no significant differences between wild type and the *ktn* mutants (*ktn1-6* and *ktn1-4*) at each indicated developmental stage (stage 8: P = 0.951, P = 0.971; stage 9: P = 0.231, P = 0.174; stage 10: P = 0.974, P = 0.882; stage 11: P = 0.765, P = 0.999; stage 12: P = 0.813, P = 0.984; stage 13: P = 0.714, P = 0.513; stage 14: P = 0.743, P = 0.924). Quantitative analysis of cell angles (C) by student *t*-test showed that, at development stages 8 to 10, the *ktn1* mutants (*ktn1-6* and *ktn1-4*)apical cone angles were similar to the wild type at indicated stage (stage 8: P = 0.075, P = 0.085; stage 9: P = 0.826, P = 0.515; stage 10: P = 0.099, P = 0.703), whereas at development stage 11 and beyond, *ktn1* mutants displayed significantly increased apical cone angles compared with the wild type (stage 11: P = 0.193, P = 0.059; stage 12: P = 0.0000823, P = 0.0000319; stage 13: P = 0.0000459, P = 0.0000383; stage 14: P = 0.0000472, P = 0.0000736). Values are given as mean ± SD of more than 300 cells of 6 petals from independent plants.

Cells from petal development stage 8 have a relatively flat surface with a 2.02-μm height, 6.18-μm width, and 143.90°cone angle on average, which have just begun to initiate conical outgrowth (Fig 2). After initiating the outgrowth from the petal epidermis, conical cells undergo both radial expansion and longitudinal elongation, with increased sharpening of apexes over the course of petal development stages 8 to 14, and a decrease in cone angles ranging from143° to 72° (Fig 2). Cells from petal development stage 9 have clearly expanded and established elongated longitudinal axes, with 4.18-μm height, 7.23-μm width, and 111.43°cone angle on average (Fig 2). After petal development stage 9, cells undergo fast anisotropic expansion with an increase in radial expansion, longitudinal elongation, and thus results in apparent conical shape. Cone angles of conical cells from stages 9 to 11 range from 111° to 72°, whereas after petal development stage 11, a slight increase in cone angle of conical cells was observed, which was caused by increased radial expansion of the cell base but relatively slow elongation of the cell’s longitudinal axis (Fig 2). The conical cell of the mature petal (stage 14) has a characteristic cone morphology with 12-μm height, 17-μm width, and 80° cone angle on average.

We next asked how KTN1 influences conical cell development at various development stages by comparing the phenotype of conical cells of wild type with the *ktn1* mutants at petal developmental stages 10–14. Measuring cell heights and cone angles at development stages 8–10 showed that the *ktn1* mutants had similar cell sizes as the wild type (Fig 2), while at developmental stage 11 and beyond, *ktn1* mutant cells displayed a decrease in tip sharpening, resulting in swollen apexes with larger cone angles compared with the wild type (Fig 2). Taken together, these results show that KTN1 plays an important role in promoting the tip sharpening of the conical cell during late development stages, thus influencing the final characteristic shape formation of the conical cell.

### 3D reconstruction of conical cells

Recent advances in 3D plant imaging have been made at both the organ scale and cellular level [42–47], which are critical for understanding morphogenesis. Having a 3D view of conical epidermal cells allows for the investigation of the contribution of spatiotemporal patterns of gene expression to 3D cell shape. To obtain a 3D surface reconstruction of conical cells, Z stacks of images from the distal regions of PI-stained petal samples were taken from the top view along their Z-axis at steps of 0.8μm to reconstruct a 3D image of conically shaped cells. As a result, we were able to obtain high-quality 3D images of conical cells (Fig 1F). We next reconstructed a 3D surface of the *ktn1* mutants and found that the 3D geometry of the mutant cells displayed increased apical isotropic expansion compared to the wild type (Fig 1F). Consistent with this result, cell morphologies visualized via scanning electron microscopy in the adaxial epidermis of wild type and the *ktn1* mutants were comparable to those observed via 3D reconstruction of Z stacks of confocal images (S4F Fig). We next performed detailed phenotype analyses of the 3D geometry of conical cells between the wild type and the *ktn1* mutants at different developmental stages. Our results showed that the 3D geometry of conical cells of the *ktn1* mutants was similar to that of wild type at the early stages (S5 Fig), but the *ktn1* mutants displayed swollen conical cell apexes after stage 11 (S5 Fig).

### ROP GTPases and SPIKE1 function in conical cell development

**Fig 3:**
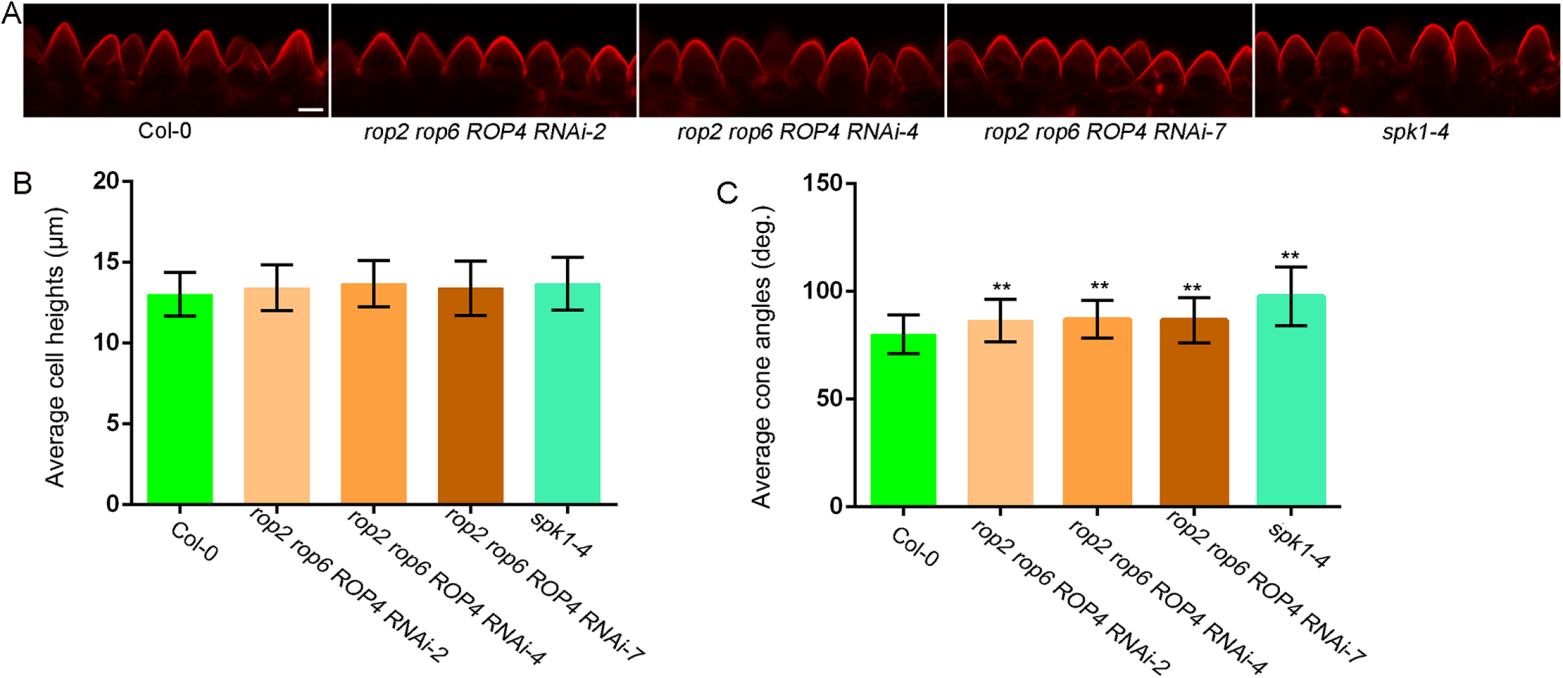
Phenotype analysis of the *rop2 rop6 ROP4RNAi* lines and the *spk1-4* mutant. (A) Representative confocal images of conical cells from stage 14 petals of wild type (Col-0), the *rop2 rop6 ROP4 RNAi* lines, and the *spk1-4* mutant. Scale bar = 10μm. (B and C) Quantitative analyses of conical cells from wild type, the *rop2 rop6 ROP4 RNAi* lines, and the *spk1-4* mutant. Confocal images of conical cells were used for quantification. For quantitative analyses of cone angles, two-way ANOVA followed by Sidak's multiple comparison test indicated a significant difference (**P<0.01) between the data sets from the *rop2 rop6 ROP4 RNAi* lines and the *spk1-4* mutant, compared with Col-0 (P = 0.00236, P = 0.0046, P = 0.00863, and P = 0.0000273, respectively). Values are given as the mean ± SD of more than 300 cells of 6 petals from independent plants.

ROP GTPases have been extensively studied for their functions in polarized cell growth [35, 48, 49], but their roles in regulating the morphogenesis of conical cells remain unknown. KTN1 is activated by ROP6 GTPase to restrict the indentation outgrowth of leaf pavement cells [28]. We investigated whether ROP GTPases contribute to conical cell development. A previous study has shown that ROP GTPases have redundant functions during petal growth, and that neither the single *rop2* or *rop6* mutant had an altered petal phenotype, whereas the *rop2 rop6 ROP4 RNAi* plants, generated by transforming a specific *ROP4 RNAi* construct into the *rop2 rop6* double mutant, had severe petal phenotypes [50]. Three *rop2 rop6 ROP4 RNAi* lines (referred to as *rop2 rop6 ROP4i*) that have been shown to have significantly decreased *ROP4* transcriptional levels [50] were used for phenotype analysis of conical cells. Our results showed that all three *rop2 rop6 ROP4 RNAi* lines had increased radial swelling of conical cell apexs but displayed no change in cell heights compared to the wild type(Fig 3); this phenotype is less severe than that of the *ktn1* mutants.

SPIKE1 (SPK1) is a dock homology region 2 (DHR2)-type ROP guanine nucleotide exchange factors (ROPGEF) and has been reported to function upstream of ROP GTPases [50, 51]. We therefore investigated the role of SPK1 during conical cell development by analyzing the *spk1-4* mutant, which was predicted to have aberrant messenger RNA splicing [50]. Our results showed that the *spk1-4* mutant also displayed swollen apexes of conical cells (Fig 3), as observed in the *rop* multiple mutants. Together, these findings suggest that SPK1, ROP GTPases, and KTN1 may function in the same pathway in the regulation of the final shape formation of conical cells.

### KTN1 regulates the spatial organization of microtubule arrays during conical cell development

**Fig 4:**
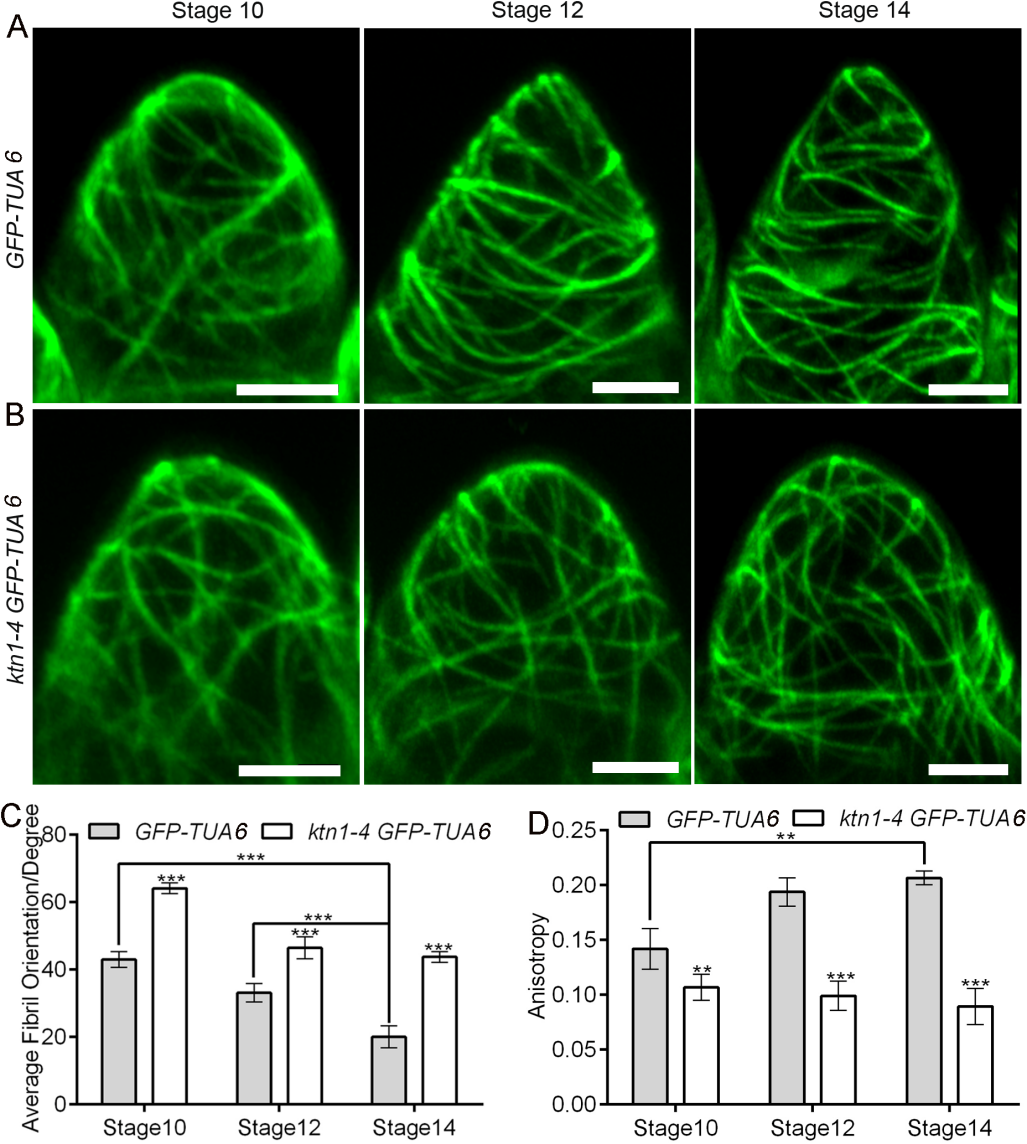
Visualization of microtubules in the serrated geometry of conical cells in wild type and *ktn1-4*. (A and B)Representative confocal images showing arrangement in the serrated geometry of conical cells from folded petals of wild type and the *ktn1-4* mutant stably expressing GFP-TUA6. Surface projections of confocal images from the adaxial epidermis of folded petals from wild type and *ktn1-4* at the indicated developmental stages. At development stage 10, wild-type cells exhibited random orientation of microtubule arrays, while at later development stages, microtubules of wild-type cells were reoriented into well-ordered transverse arrays. In contrast, the *ktn1-4* mutant cells displayed random microtubules arrays throughout petal development stages. Scale bars = 5μm. (C and D) Quantitative analyses of the average orientation and anisotropy of microtubules in wild-type and *ktn1-4* conical cells. FribrilTool, an ImageJ plug-in, was used for quantification of the orientation angle (Average Fibril Orientation) and the anisotropy of microtubules in a given region of interest. Anisotropy values range from 0 to 1. 0 indicates pure isotropy, and 1 represents pure anisotropy. For quantitative analyses of the average fibril orientation (C), two-way analysis of variance (ANOVA) followed by Sidak's multiple comparison test indicated a significant difference (***P<0.001)between the data sets from stage 10 and stage 12 *GFP-TUA6* line compared with stage 14*GFP-TUA6* line (P = 0.0000042 and P = 0.0000273, respectively), and between the data sets from *GFP-TUA6* line compared with the *ktn1-4 GFP-TUA6* line (for stage 10, P = 0.000013, for stage 12, P = 0.00023, for stage14, P = 0.0000227).For quantitative analyses of the anisotropy of microtubules (D), two-way ANOVA followed by Sidak's multiple comparison test indicated a significant difference (**P<0.01 and ***P<0.001)between the data sets from stage 10 *GFP-TUA6* line compared with stage 14 *GFP-TUA6* line (P = 0.00268), and between the data sets from *GFP-TUA6* line compared with the *ktn1-4 GFP-TUA6* line (for stage 10, P = 0.00128, for stage 12, P = 0.000004227, for stage14, P = 0.00000458).Values are given as the mean ± SD of more than 50 cells of 6 petals from independent plants.

**Fig 5:**
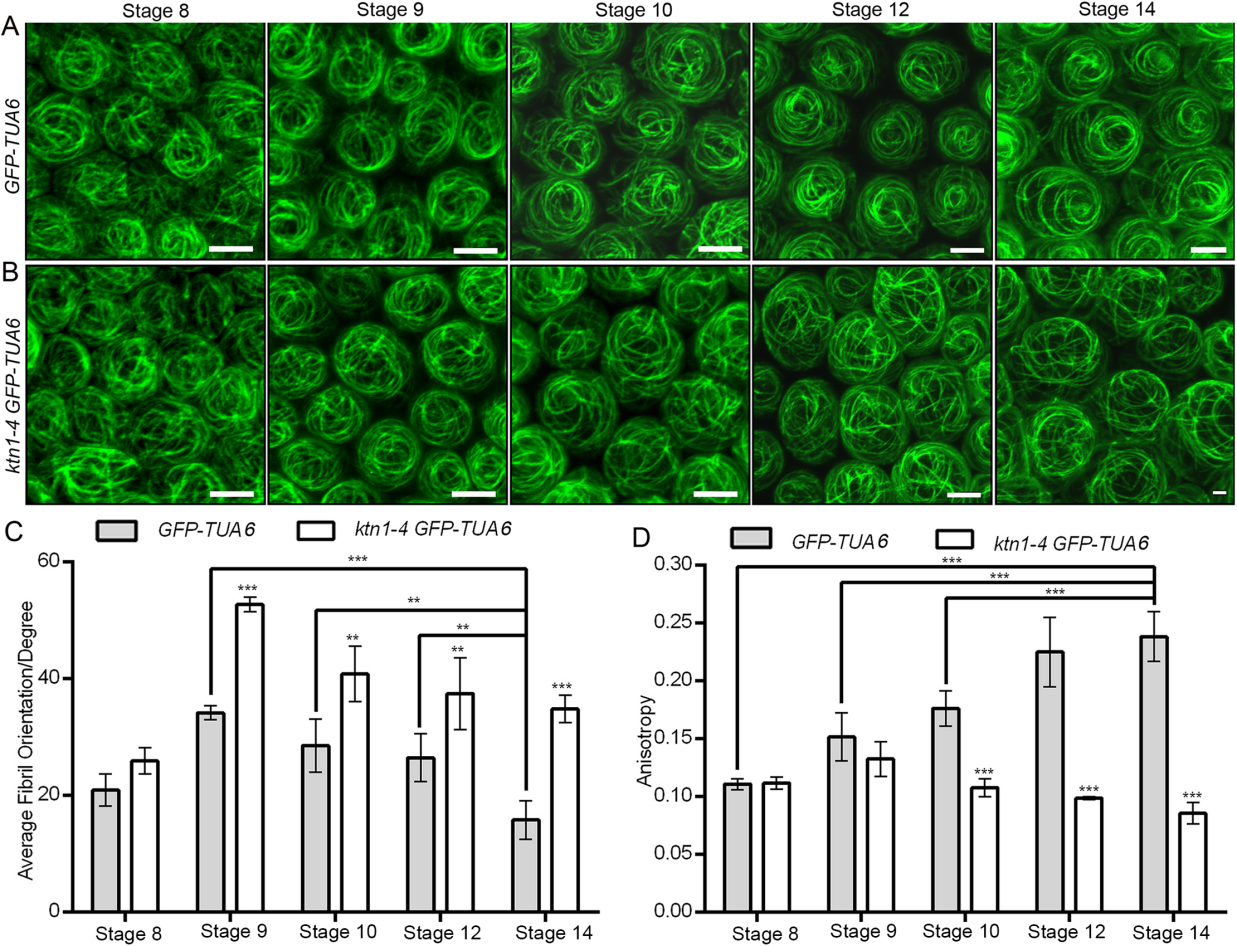
KTN1 induces microtubule reorientation into well-ordered circumferential arrays at late developmental stages. (A and B)Visualization of microtubules in conical cells of both wild type and the *ktn1-4* mutant stably expressing GFP-TUA6, respectively. Confocal images of surface projections from the top view of wild-type (A) and *ktn1-4* (B) petal adaxial epidermis at the indicated developmental stages. At early developmental stages, wild-type cells exhibited random orientation of microtubules arrays, but became increasingly well-ordered circumferential orientation of microtubules arrays after stage 10. By contrast, the *ktn1-4* mutant cells had random microtubules arrays throughout petal development stages 8–14. Scale bars = 5μm. (C and D) Quantitative analyses of the average orientation and anisotropy of microtubules in wild-type and *ktn1-4* conical cells. FribrilTool, an ImageJ plug-in, was used for the quantification of the orientation angle (Average Fibril Orientation) and the anisotropy of microtubules in a given region of interest of the wild type and *ktn1-4*. For quantitative analyses of the average fibril orientation (C), two-way ANOVA followed by Sidak's multiple comparison test indicated a significant difference (**P<0.01 and***P<0.001)between the data sets from stage 9, stage 10, and stage 12 *GFP-TUA6* line, compared with stage 14 *GFP-TUA6* line (P = 0.00000463,P = 0.004896, and P = 0.00481, respectively), and between the data sets from *GFP-TUA6* line compared with the *ktn1-4 GFP-TUA6* line (for stage 9, P = 0.0000598, for stage 10, P = 0.001601, for stage12, P = 0.002649, for stage 14, P = 0.0000169). For quantitative analyses of the anisotropy of microtubules (D), two-way ANOVA followed by Sidak's multiple comparison test indicated a significant difference (***P<0.001) between the data sets from stage 8, stage 9, and stage10*GFP-TUA6* line compared with stage 14 *GFP-TUA6* line (P = 0.00000418, P = 0.000000194, and P = 0.0000861, respectively), and between the data sets from *GFP-TUA6* line compared with the *ktn1-4 GFP-TUA6* line (for stage 10, P = 0.000000359, for stage 12, P = 0.00000126, for stage14, P = 0.0000000791). Values are given as the mean ± SD of more than150 cells of 6 petals from independent plants.

To investigate the cellular mechanism by which KTN1 affects the morphogenesis of conical cells, we monitored microtubule organization patterns at different stages of conical cells via live imaging of *A*. *thaliana* plants expressing green fluorescent protein (GFP)-tagged α-tubulin 6 (GFP-TUA6) [52] in the control and the *ktn1-4* mutant. We observed the organization of microtubule arrays in the serrated shape of conical cells from folded petals. We used circular statistics to collect quantitative data by quantifying the orientation angle and anisotropy of microtubules via FribrilTool [53], which was used for the quantification of the orientation and anisotropy of fibrillar structures in a given region of interest (ROI) from raw images using the software ImageJ. The average fibril orientation is defined by the circular average of the tangent direction in the ROI region, and the circular variability of tangent directions defines the score of the fibril array anisotropy [53]. Our results showed that, at the early developmental stages, wild-type conical cells observed from folded petals exhibited a network of microtubule arrays that were randomly oriented (Fig 4A, 4C and 4D), whereas at later development stages, conical cells with sharpening apexes were associated with transverse microtubule arrays (Fig 4A, 4C and 4D). By contrast, *ktn1-4* mutant cells had randomly oriented microtubule arrays at both the early and late developmental stages (Fig 4B–4D). We also observed microtubule arrays via maximum projections of Z stacks from the top view of non-folded petals, and found that wild-type cells exhibited microtubule arrays with random orientation at early stages but well-ordered circumferential orientations at later stages (Fig 5A, 5C and 5D). By contrast, the *ktn1-4* mutant cells had random microtubule arrays at both early and late developmental stages (Fig 5B–5D).

Furthermore, we visualized microtubule arrays configuration via 3D reconstruction of conical cells from the top view of non-folded petals. We examined patterns of microtubule arrangements in detail throughout development stages 8–14. Similarly to the observation in serrated conical cells from folded petals, microtubule arrays in wild-type conical cells were randomly oriented at the early developmental stages (S6A Fig), and then became increasingly ordered during later development stages (stages 12 to 14) (S6A Fig). Highly ordered transverse microtubule rings encircling wild-type conical cells were found at stage 14 (S6A Fig). By contrast, loss of KTN1 function prevented random microtubule networks from shifting into transverse microtubule rings encircling conical cells (S6B Fig).

We next examined microtubule organization patterns in both petal abaxial epidermal blade cells and claw cells; these epidermal cells have flat shape [50]. Cortical microtubule arrays in wild-type abaxial blade cells were randomly oriented and a few transverse microtubules were associated with indentation regions of these cells, and microtubules in the *ktn1-4* abaxial blade cells were disordered compared with the wild type (S7A and S7B Fig). By contrast, microtubule arrays in wild-type petal claw cells were highly parallel and transversely oriented to the axis of cell elongation (S7C and S7D Fig). Microtubules in the *ktn1-4* petal claw cells were organized into “net-like” arrays in which microtubules display no particular alignment (S7C and S7D Fig). Taken together, KTN1-dependent microtubule organization patterns appear to differ between cell types, which is important for shaping diverse plant epidermal cells.

### Microtubules but not actin filaments play major roles in controlling the final shape formation of conical cells

**Fig 6:**
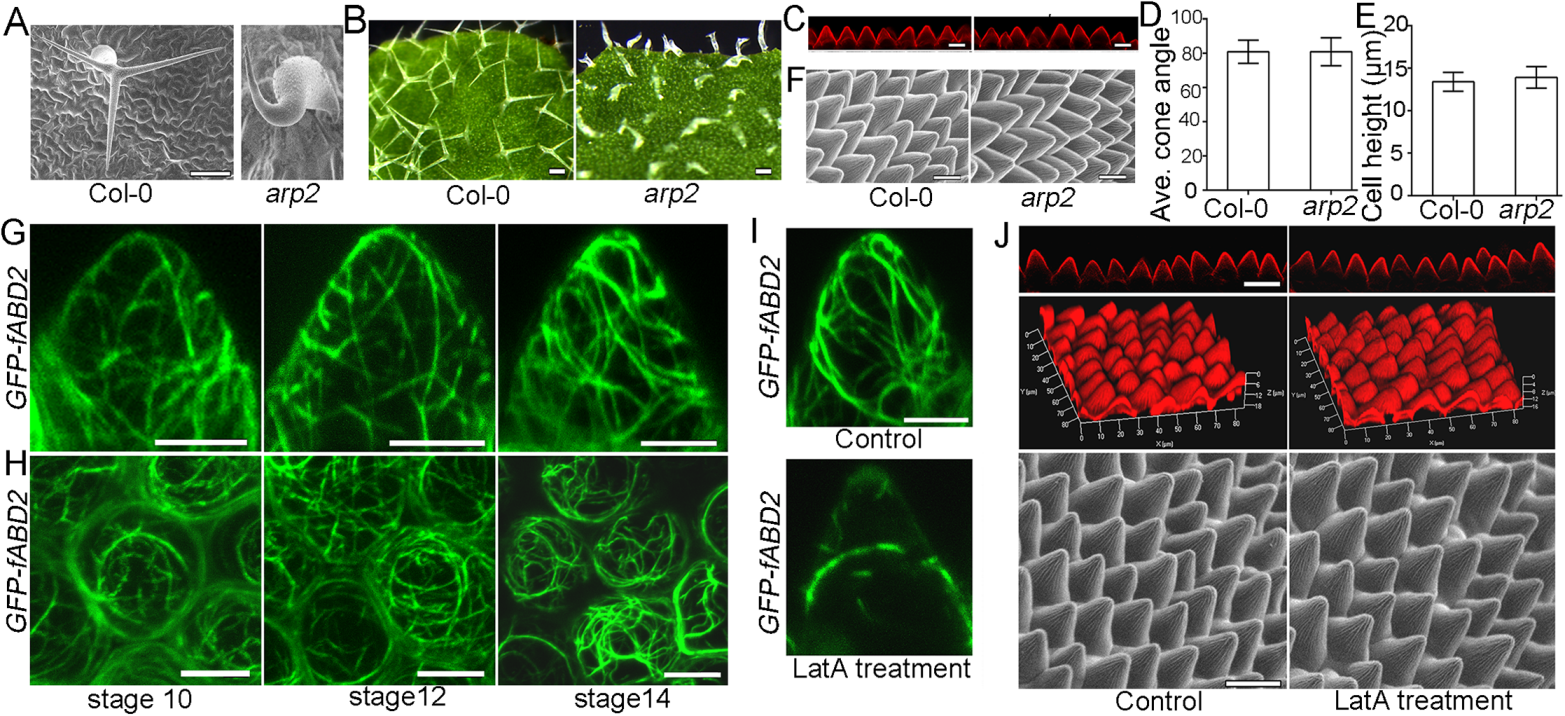
Actin is not required for conical cell shaping. (A)Representative images via a TM-3030 table-top scanning electron microscope (Hitachi) view of leaf trichomes in wild type and the *arp2* mutant. Scale bar = 100μm. (B) Representative images via stereo microscope view of leaf trichomes in wild type and the *arp2* mutant. Scale bars = 100μm. (C) Confocal images of conical cells from folded petals of wild type and the *arp2* mutant. Note that there is no obvious difference between wild type and the *arp2* mutant. Scale bars = 10μm. (D and E) Quantification analysis of conical cell phenotypes. One-way ANOVA followed by Sidak's multiple comparison test indicated no significant difference between the data sets from the *arp2* mutant compared with Col-0 (for D, P = 0.964; for E, P = 0.1344). Values are given as the mean ± SD of more than 100 cells of 5 petals from independent plants. (F)Representative images via a TM-3030 table-top scanning electron microscope view of conical cells. Note that there is no obvious difference between wild type and the *arp2* mutant. Scale bars = 10μm. (G and H) Actin organization patterns in conical cells. Representative confocal images showing actin organization patterns in the serrated geometry of conical cells from the GFP-fABD2 marker line (G). Confocal images of surface projections from the top view of non-folded petals from the GFP-fABD2 marker line (H). Scale bars = 5μm. (I) Application of latrunculin A showing depolymerization of F-actin. Stage 7 floral buds of the GFP-fABD2 marker line were immersed in a solution containing100 μg/mL latrunculin A for 5 min treatment. To prevent repolymerization of the F-actin, the same treatment was repeated 24 h later for another two times. Scale bar = 5μm. (J) Application of latrunculin A had no effect in conical cell shape by live-confocal imaging and scanning electron microscope analyses. Scale bars = 20 μm.

Previous results have shown that actin filaments play pivotal roles during plant cell shape formation and that trichome mutants with defects in the plant actin-related protein ARP2/3 complex serve as an excellent system for the study of actin-dependent cell morphogenesis [54–56]. We investigated whether the *arp2* mutant showed defects in conical cell shape. Consistently with previous reports, we found that *arp2* mutant plants had swollen trichomes, with stunted branch outgrowth (Fig 6A and 6B). However, the conical cell shape in the *arp2* mutant was similar to the wild type (Fig 6C–6F).

A previous report has shown that both transverse microtubule rings and the cortical actin cap are required for branch tip sharpening during leaf trichome development in wild type, and that these cytoskeletal organization patterns are disrupted in the loss-of-function mutant of KCBP (kinesin-like calmodulin-binding protein) that has trichomes with swollen tips [57]. We next investigated whether the *kcbp* mutant showed tip defects in conical cells. Consistently with previous reports, we found that the *kcbp* mutant trichomes with two branches showed swollen branch tips (S8 Fig); however, the shape of conical cells was similar to the wild type (S8 Fig). We next investigated whether the *ktn1-4* mutant showed defects in trichome branch tips. Strikingly, we found that the *ktn1-4* mutant had two*-*branched leaf trichomes displaying no swollen tips compared with the wild type (S9 Fig). Taken together, our findings suggest that KTN1-dependent microtubule organization is involved in the tip sharpening of conical cells but is not essential for the branch tip sharpening during leaf trichome development.

We next examined the organization patterns of actin filaments. Live-cell imaging of the actin marker GFP-fABD2 line [58] showed that actin filaments displayed a disordered array in conical cells throughout development stages 7–14 (Fig 6G and 6H and S10 Fig). In addition, actin filaments cables became thick and dense at late stages during conical cell development (Fig 6G and 6H). Surprisingly, over the course of conical cell development, we could not observe an apical cap of actin filaments (Fig 6G and 6H and S10 Fig), which is a typical feature of diverse cell types, such as root hairs, leaf trichomes, and zygotes [59–61].

To examine the respective roles of microtubules and actin, we investigated how their inhibitors influenced conical cell development. We depolymerized the microtubules and actin with the use of the microtubule polymerization inhibitor (oryzalin) and the actin polymerization inhibitor [latrunculin A (LatA)], and analyzed their influence on cell morphologies. Both the filamentous pattern of the microtubule and the actin signal diffused when oryzalin or LatA were applied (S11A Fig and Fig 6I), confirming that these inhibitors were efficient in our experimental setup.

**Fig 7:**
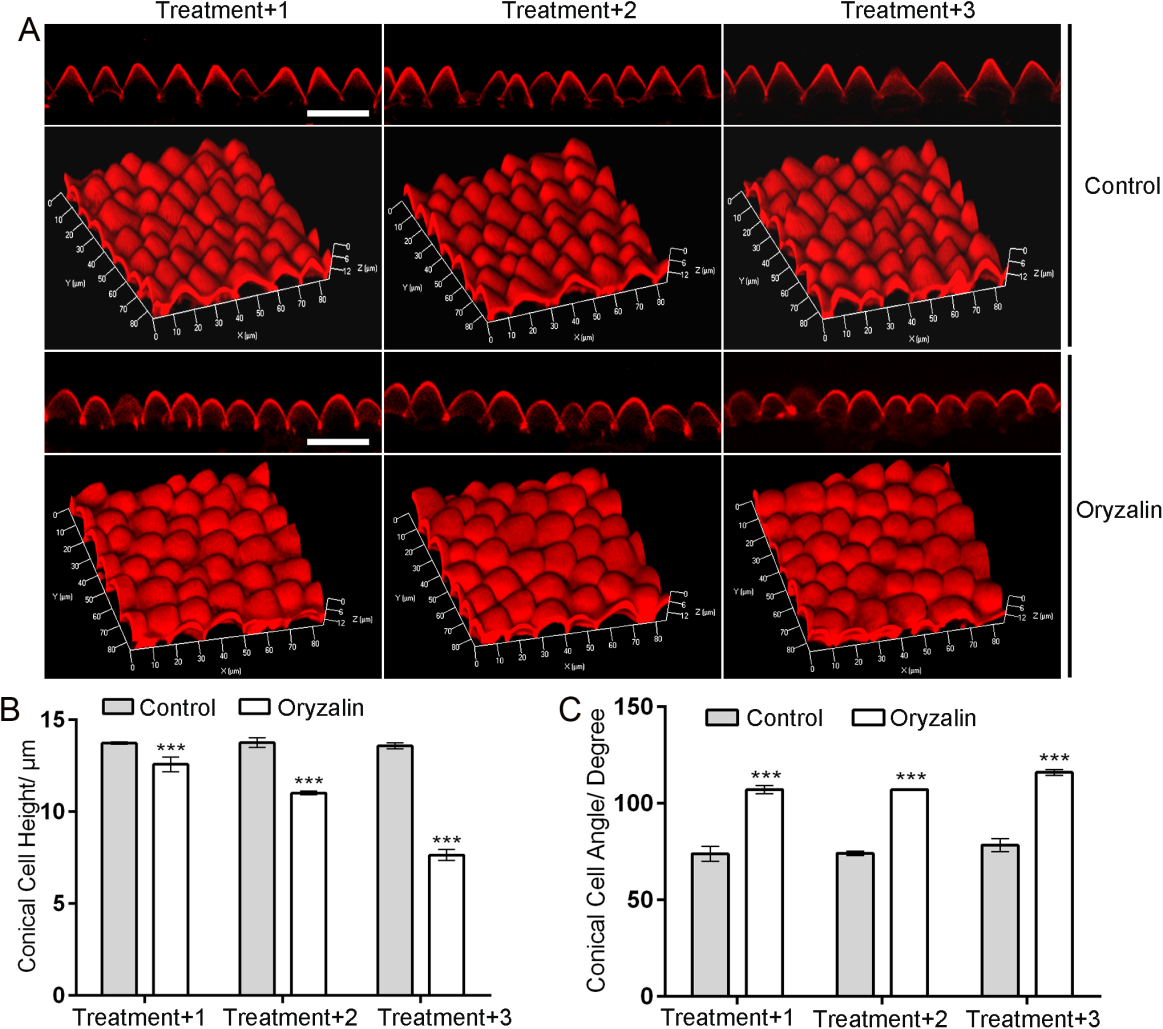
Depolymerizing microtubules causes increased expansion of conical cell apexes. (A) Example confocal images of wild-type conical cells with or without oryzalin treatment. Floral buds at stage 8 that had flat epidermal cells were immersed in a solution containing 30 μg/ml oryzalin for 5-min treatment. To prevent repolymerization of the microtubules, the same treatment was repeated 24 h later for another two times. Three situations were investigated, where the floral buds were treated by oryzalin or DMSO control for one time (Treatment+1), two times (Treatment+2), or three times (Treatment+3), respectively. Application of DMSO solution was set as control treatment. The mature petals at stage 14 were used for cellular phenotype analyses. Scale bars = 25 μm. (B and C)Quantitative analyses of wild-type conical cells with or without oryzalin treatment. For quantitative analyses of cell heights (B), two-way ANOVA followed by Sidak's multiple comparison test indicated a significant difference (***P<0.001)between the data sets from the control compared with oryzalin treatment (For treatment+1, P = 0.0000000254, for treatment+2, P = 0.00000016, treatment+3, P = 0.00000068). For quantitative analyses of cell heights (B), two-way ANOVA followed by Sidak's multiple comparison test indicated a significant difference (***P<0.001)between the data sets from the control compared with oryzalin treatment (For treatment+1, P = 0.0000000254, for treatment+2, P = 0.00000016, treatment+3, P = 0.00000068). For quantitative analyses of cell angles (B), two-way ANOVA followed by Sidak's multiple comparison test indicated a significant difference (***P<0.001)between the data sets from the control compared with oryzalin treatment (For treatment+1, P = 0.000000568, for treatment+2, P = 0.000000421, treatment+3, P = 0.00000014). Values are given as the mean ± SD of more than 300 cells of 6 petals from independent plants.

We next examined the effects of these inhibitors on conical cell morphologies. Floral buds before stage 8 were immersed in a solution containing 30 **μ**g/ml oryzalin for 5 min. To prevent repolymerization of the microtubules, the same treatment was repeated once or twice 24 h later. Three situations were investigated, where the oryzalin treatment was applied one, two, and three times. To observe the geometry of cells, cell phenotypes of mature petals at stage 14 after the oryzalin treatment were analyzed. In contrast to the control treatments, the application of oryzalin had significant effects on cell morphology, causing increased radial swelling at the tip of conical cells with increased cone angle and reduced cell height (Fig 7). In addition, visualization of conical cells via both 3D reconstruction and scanning electron microscope further confirmed that oryzalin treatment caused the isotropic growth of the tip of the conical cell (Fig 7 and S11B Fig). By contrast, treatment with LatA did not cause significant alterations on conical cell morphology (Fig 7J), suggesting that actin filaments may not play a major role in the final shape formation of conical cells. Furthermore, depolymerizing actin filaments by treatment with LatA had no effect on the configuration of transverse ring of microtubules (S12 Fig), suggesting that actin is not required for organizing microtubules during conical cell development.

## Discussion

In this study, we presented a confocal laser scanning microscopy-based imaging technique that can be used for the quantitative study of conical cells' geometry in *A*. *thaliana*. Our results showed that petal folding had no effect on the geometry of conical cells by comparing the serrated images generated by the side view of conical cells from petal folding with images of histological sections of fixed samples. In addition, petal folding in our experimental condition (with gently folding the petal), allowing for the side view of conical cells, did not alter microtubule organization patterns. Therefore, the use of the live-confocal imaging technique will open exciting new avenues of research to study the genetic and molecular mechanisms controlling the final shape formation of conical cells.

Although the mechanisms underlying microtubule organization have been extensively studied in diverse cell types [24, 25, 57, 62–64], the configuration of microtubule arrays in petal conical cells remains unknown. Live-cell imaging of GFP-TUA6 to study microtubule organization in *A*. *thaliana* petal conical cells suggests that in the early stages, microtubule arrays are randomly oriented, which results in isotropic expansion of conical cells, and that at later stages, microtubule arrays are reoriented into well-ordered circumferential arrays, which leads to an increase in tip sharpening of the conical apex over the course of the conical cell development, and thus forming the final characteristic conical-shaped cell with an average cone angle of 80°. We propose that conical cell is an excellent model system for investigating the spatio-temporal orientation of microtubule arrays. Therefore, conical cell shaping could become a valuable complement to other more popular systems used to study cell shape, such as leaf pavement cells [35].

Our results showed that the microtubule organization in conical cells appears essentially identical to that observed in leaf trichomes, whose morphogenesis is cooperatively regulated by microtubules and actin filaments [57, 60]. By combining genetic and pharmacological experiments using specific inhibitors for different cytoskeletal elements, our results suggested that microtubules but not actin filaments play pivotal roles in conical cell development. Strikingly, a cap of actin filaments, which is frequently found in diverse cell types in plants, such as root hairs, leaf trichomes, pollen tubes and zygotes [59–61], was not observed in the growth apexes of conical cells during cell development. A previous report has shown that the actin-related protein ARP2/3 complex drives an actin meshwork that functions within a tip-localized, microtubule-depleted region to regulate cell wall anisotropy and leaf trichome morphogenesis [65]. Our findings showed that the *arp2* mutant and the *kcbp* mutant showed normal conical cell shape and that depolymerizing of actin filaments had no effect on the configuration of transverse ring of microtubules in mature conical cells, suggesting that actin filaments is not critical for conical cell development. Thus, we propose that distinct mechanisms are required for the cytoskeletal control of leaf trichome morphogenesis and conical cell development. Given that actin appears more important for the initial outgrowth, while microtubules are more important for later elongation during leaf trichomes and root hairs development, we cannot exclude the roles of actin filaments in the initial conical outgrowth during conical cell development.

Our findings showed that KTN1 mainly functions at late development stages to generate parallel circumferential microtubule arrays, which may lead to the tip sharpening of the conical cell apex over the course of development, probably through affecting cell wall patterns [20–22]. Previous studies have shown that the activity of KTN1 is precisely controlled by two microtubule-associated proteins in *A*. *thaliana*: RIC1 and SPR2 [28, 30]. RIC1, an effector of ROP6 GTPase, activates KTN1 to promote parallel ordering of microtubule arrays in leaf pavement cells [28, 29]. SPR2 accumulates at the microtubule crossover sites to prevent severing by KTN1, allowing randomly oriented microtubule arrays to persist [30], suggesting that SPR2 may also function to activate KTN1 during conical cell development. It is more likely that SPR2 is more mobile in conical cells (especially during later development stages) as found in leave petiole cells, which is required for the activation of KTN1 during the tip sharpening of conical cells at later stages.

Our results showed that SPK1 and ROP GTPases function in the regulation of the final characteristic shape formation of conical cells, suggesting that SPK1 and ROP GTPase may be required for the spatio-temporal activation of KTN1 during conical cell development. Given that our results showed that both SPK1 and ROP GTPases (ROP2, ROP4 and ROP6) had more modest effects on cone angle of conical cells in comparison with KTN1, it is possible that other ROPs may also participate in conical cell development and that other novel signaling components that need to be identified in future studies are also required for the activation of KTN1. Future studies should aim to investigate the mechanisms by which KTN1's activity is spatio-temporally regulated during conical cell development. In addition, it will be important to examine the contributions of patterns of cell wall stiffness to the morphogenesis of conical cells.

## Materials and methods

### Plant materials and growth conditions

*A*. *thaliana* ecotypes Col-0 and WS were used in this study. The *ktn1-4* (SAIL_343_D12), *arp2* (SALK_003448C), and *kcbp-1* (SALK_017886C) were obtained from the Arabidopsis Biological Resource Centre. Seeds were sterilized, plated on Murashige and Skoog medium agar petri dishes supplemented with 1% (w/v) sucrose, and germinated. Plants were grown in a growth room at 22°C under 16-hr light/8-hr dark cycles.

### Visualization of conical cells by confocal laser scanning microscopy

To visualize the geometry of conical cells, *A*. *thaliana* petals from flowers at stages 10 to 14 were carefully dissected. We developed a rapid imaging method for observing the serrated shape of conical cell using fluorescent microscopy. Conical cells protrude outwards from the plane of the adaxial epidermis, so the lateral cell walls that are not in the plane of the focal axis of the confocal microscope cannot be observed from the top view of a faced-up petal sample. To make a side view of the conical cells, petal blades were transversally folded back to expose the adaxial interface to the fold, thus allowing observation of the serrated shape of conical cells. The petal samples were put onto a micro slide and then were folded back. A cover slide was slightly put on the samples. Then, a staining solution containing 10 μg/ml propidium iodide was added through the cover slide edge and samples were incubated for at least 10 min. Then, the serrated shape of cones was visualized at the position of the folded interface by fluorescent microscopy. This fast observation of conical cells enables high-throughput genetic screening for mutants with abnormal conical cell shapes. For quantifications of cell shape, samples were imaged with a Zeiss LSM 880 confocal laser scanning microscope. Z stacks of optical sections were taken and projected on a plane to generate a quantifiable serrated geometry of conical cells.

For imaging 3D geometry of conical cells, Z stacks of confocal images from the distal regions of PI-stained petal samples from the indicated development stages were taken from the top view along their Z axis at steps of 0.8 μm, and were used to reconstruct the 3D images using Zeiss LSM 880 software.

For observations of cortical microtubules, petal samples stably expressing GFP-Tubulin6 were imaged by confocal scanning, and serial optical sections were taken at 0.6 μm increments with a 63× oil lens, and then projected on a plane (i.e. maximum intensity), or were used to reconstruct the 3D images using Zeiss LSM 880 software for 3D view of the configuration of microtubule arrays.

### Mutant screening

Approximately 5,000 seeds of wild-type Col-0 were mutagenized using ethyl methane sulfonate. M_2_ seeds were harvested from self-fertilized M_1_ plants individually, and M_2_ lines were screened for altered phenotypes of petal conical cells in comparison with wild-type Col-0. Candidate mutants were backcrossed to Col-0 three times before further phenotype analyses.

### DNA constructs and plant transformation

For complementation experiments, the promoter region of *KTN1* gene was amplified by PCR from Col-0 genomic DNA using the following primers: KTN1Pro-EcoR1-F:5’ GCGAATTCTTTCTTGTATCCAATAAAGTGACCAC 3’, *KTN1Pro-Sac1-R*: 5’GCGAGCTCAAAACAAAATCAAGGGTTCCGA 3’. And the *KTN1* coding sequence was amplified by PCR from Col-0 total mRNA reversed cDNA using the following primers:KTN1CDS-Sac1-F: 5’GCGAGCTCATGGTGGGAAGTAGTAATTCG 3’, KTN1CDS-Sal1-R: 5’GCGTCGACTTAAGCAGATCCAAACTCAGAG3’.

The resulting DNA fragments were cloned into the pCambia1300 vector. The resulting *pKTN1*::*KTN1* was introduced into the *ktn1-6* mutant by *Agrobacterium tumefaciens*-mediated floral dip transformation.

### Chemical treatments

For oryzalin treatment, a 30 mg/ml oryzalin (Sigma, 36182) stock solution dissolved with DMSO was prepared (working solution: 30 μg/ml oryzalin containing 0.01% silwet L-77). Floral buds at stage 8 that had flat epidermal cells were immersed in the solution containing 30 μg/ml oryzalin for 5-min treatment. To prevent repolymerization of the microtubules, the same treatment was repeated 24 h later for twice. For Latrunculin A treatment, a 100 μg/mL Latrunculin A (Sigma, L5163) stock solution dissolved with DMSO was prepared (working solution: 0.5 μg/mL Latrunculin A containing 0.01% silwet L-77). Floral buds at stage 8 that had flat adaxial epidermal cells were immersed in the Latrunculin A working solution for 5-min treatment. To prevent repolymerization of the microfilaments, the same treatment was repeated twice24 h later.

### Scanning electron microscopy

To observe petal epidermal cells, detached petal samples were directly observed with a TM-3000 table-top scanning electron microscope (Hitachi)equipped with a cool stage.

### Quantification methods

For the quantification of the geometric parameters of conical cells, cell heights, cell angles, and gap heights were manually measured using ImageJ software. More than 300 cells of 6 petals from independent plants were measured. For the quantification of the average orientation and anisotropy of microtubule arrays in wild-type and *ktn1-4* conical cells. FribrilTool [53], an ImageJ plug-in, was used for quantification of the orientation angle and the anisotropy of microtubule arrays in a given region of interest of the wild type and *ktn1-4*. Anisotropy values range from 0 to 1. 0 indicates pure isotropy, and 1 represents pure anisotropy.

### Statistical analysis

Statistical analyses were performed using student’s t test, and one-way or two-way ANOVA. Data were represented as the mean ± SEM from at least three independent experiments. Not significant P > 0.05, 0.01 <*P < 0.05, 0.001<**P < 0.01, ***P < 0.001.

### Accession numbers

Sequence data from this article can be found in the Arabidopsis Genome Initiative or GenBank/EMBL databases under the following accession numbers: AT1G80350 (*KTN1*), AT1G20090 (*ROP2*), AT1G75840 (*ROP4*), AT5G65530 (*ROP6*), AT4G16340 (*SPK1)*, AT3G27000 (*ARP2*), AT5G65930 (*KCBP*).

## Supporting information

S1 Fig Methods for observation of conical cells. (A) A wild-type mature *A*. *thaliana* flower (stage 14) for observation of adaxial epidermal cell shape. The square area is visualized by confocal. (B) Confocal imaging analysis of petal adaxial epidermal cells from the top view of a petal sample. Scale bar = 10μm.(C) A folded back petal for observation of the serrated shape of conical cells from the side view of conical cells. The square area of the folded back petal allows for the side visualization of the conical cells by confocal. (D and E) Example confocal images of conical adaxial epidermal cells and flat abaxial epidermal cells from petals of *Arabidopsis*. Scale bars = 10μm. For the observation of conical adaxial epidermal cells and flat abaxial epidermal cells, adaxial epidermis and abaxial epidermis are folded back, respectively. (F and G) Representative images via a TM-3000 table-top scanning electron microscope view of *A*. *thaliana* petal adaxial epidermis and abaxial epidermis. The petal adaxial epidermis has conical-shaped cells (F), while the abaxial epidermis has flat-shaped cells (G). Scale bars = 10μm. (H) A cartoon depicting how cell heights, indentation heights, and cone angles are manually measured using the ImageJ software.
(TIF)

S2 Fig Toluidine-blue stained cross section of a mature wild-type petal and quantification of conical cells. (A) A representative image of toluidine-blue stained cross section of a mature *A*. *thaliana* wild-type petal. Scale bar = 20μm. (B–D) Quantitative analyses of the geometry of wild-type conical cells. Propidium iodide-stained folded petals were visualized by confocal microscope, and toluidine-blue stained cross sectional petals were observed by optical microscope. Cell heights (B), cell widths (C), and cone angles (D) were quantified from the images made by these two imaging methods. Quantification data shows no significant differences of the geometry of conical cells from the images made by these two imaging methods [student’s *t*-test, P = 0.092 (B), P = 0.078 (C), and P = 0.124 (D)]. Values are given as the mean ± SD of more than 300 cells of 6 petals from independent plants.
(TIF)

S3 Fig Complementation of the *ktn1-6* mutant. (A) The identification of the *ktn1-6* and *ktn1-4* mutants. (B) Identification of the *ktn1-6* mutation by dCAPS1 marker. The *ktn1-6* mutation disrupts the cleavage site of SpeI. (C and D) Complementation of the *ktn1-6* mutant. Representative confocal images of the geometry of conical cells from wild type, *ktn1-6*, and the *ktn1-6* complementation line (C). Complementation of *ktn1-6* by transforming *pKTN1::KTN1* into the *ktn1-6* plants. More than ten complementation lines were obtained and one representative transgenic line(*ktn1-6 COM#25*) displaying similar conical cell shape to the wild type is shown. Scale bar = 10μm. Quantitative analyses of the geometry of conical cells (D). The average cell height and cell angle from the complementation line were similar to those of the wild type (student *t*-test, P = 0.672, P = 0.723). Values are given as the mean ± SD of more than 280 cells of 5 petals from independent plants.
(TIF)

S4 Fig The shape of the basal parts of conical cells and TM-3030 table-top scanning electron microscope view of adaxial epidermis. (A) Representative confocal images of the basal parts of conical cells. Mature petals at development stage 14 were used for imaging analysis of adaxial epidermal cells from the top view via confocal microscopy. The *ktn* mutants' cells showed similar hexagonal base to the wild type. Scale bar = 10μm. (B–E)Analyses of cell length (B), cell width (C), cell index (D), and cell area (E) showed that the hexagonal basal sizes of conical cells of the *ktn* mutants were similar to those of the wild type. Values are given as the mean ± SD of more than 200 cells of petals from independent plants. (F) Representative images via a TM-3000 table-top scanning electron microscope view of adaxial epidermis. The *ktn1* mutants displayed increased isotropic apical expansion of conical cells compared with the wild type. Three independent experiments were conducted and showed similar results. Scale bar = 10μm.
(TIF)

S5 Fig 3D reconstructions of conical cells of wild type, *ktn1-6*, and *ktn1-4* from various development stages. Representative images of 3D geometry of conical cells at the indicated developmental stages from wild type and the *ktn* mutants. Z stacks of confocal images from the distal regions of PI-stained petal samples from various developmental stages were taken from the top view along their Z axis at steps of 0.8 μm to reconstruct the 3D images.
(TIF)

S6 Fig 3D reconstructions of wild-type and *ktn1-4* conical cells expressing GFP-TUA6. (A and B) 3D reconstructed microtubule configuration in wild-type (A) and the *ktn1-4* mutant (B) conical cells stably expressing GFP-TUA6 at the indicated developmental stages.
(TIF)

S7 Fig Microtubule organization patterns in abaxial petal blade epidermal cells and petal claw cells in wild type and *ktn1-4*. (A and C) Representative confocal images showing microtubule arrangement in petal abaxial blade epidermal cells (A) and adaxial petal claw cells (C) from both wild type and the *ktn1-4* mutant stably expressing GFP-TUA6. Surface projections of confocal images from the abaxial epidermis of the non-folded petals. Scale bar = 10 μm. (B and D) Quantitative analysis of the average fibril orientation in abaxial blade epidermal cells (B) and adaxial petal claw cells (D) from wild-type and *ktn1-4* petals. FribrilTool, an ImageJ plug-in, was used for quantification of the orientation angle. One-way ANOVA followed by Sidak's multiple comparison test indicated a significant difference (*P<0.05 and ***P<0.001) between the data sets from the *GFP-TUA6* line compared with the *ktn1-4 GFP-TUA6* line [P = 0.02302 (B), and P = 0.000000322 (D)]. Values are given as the mean ± SD of more than 100 cells of 3 petals from independent plants.
(TIF)

S8 Fig Phenotypic analyses of leaf trichomes and petal conical cells in wild type and the *kcbp-1* mutant. (A) Representative images via stereo microscope view of leaf trichomes in wild type and the *kcbp-1* (*zwi*) mutant. Scale bar = 0.5 cm. (B) Example images of petal conical cells from wild type and the *kcbp-1* mutant. Note that there is no obvious difference between wild type and the mutant. Scale bars = 10μm. (C)Representative images via a TM-3030 table-top scanning electron microscope view of conical cells from wild type and the *kcbp-1* mutant. Note that there is no obvious difference between wild type and the mutant. Scale bars = 10μm.
(TIF)

S9 Fig Representative images via a TM-3030 table-top scanning electron microscope view of leaf trichomes. The *ktn1-4* mutant has two-branch trichomes, displaying no swollen tips compared with the wild type. Scale bars in top and bottom panel represent 400 μm and 100 μm, respectively.
(TIF)

S10 Fig 3D reconstructions of wild-type and *ktn1-4* conical cells stably expressing GFP-fABD2. 3D reconstructed actin filaments configuration in wild-type the *ktn1-4* conical cells stably expressing GFP-fABD2 at the indicated developmental stages.
(TIF)

S11 Fig Depolymerization of microtubules causes increased isotropic cell expansion in conical epidermal cells. (A) Application of oryzalin showing depolymerization of microtubules. Stage 7 floral buds of the GFP-TUA6 transgenic line were immersed in a solution containing 30 μg/ml oryzalin for 5 min. To prevent repolymerization of the microtubules, the same treatment was repeated 24 h later for another two times. Microtubules in conical cells from petal development stage 14 were observed. Application of oryzalin could cause depolymerize microtubules in conical cells and increased isotropic cell expansion in the GFP-TUA6 transgenic line. Three independent experiments were conducted and showed similar results. Scale bar = 2 μm. (B) Representative images via a TM-3030 table-top scanning electron microscope (Hitachi) view of wild-type conical cells. Application of oryzalin caused increased isotropic cell expansion in conical epidermal cells. Three independent experiments were conducted and showed similar results. Scale bar = 10μm.
(TIF)

S12 Fig Depolymerizing of F-actin has no effect on microtubule organization. (A and B) Depolymerizing of F-actin by treatment with LatA had no effect on the configuration of transverse ring of microtubules in the *GFP-TUA6* conical cell. Stage 14 flower of the *GFP-TUA6* transgenic line were immersed in a solution containing 0.5 μg/ml latrunculin A for 15-min treatment. Scale bar = 2 μm. (C)Quantification of anisotropy of microtubule arrays showed that there was no significant difference between control and LatA treatment (One-way ANOVA, P = 0.652). Values are given as the mean ± SD of 10 cells. (D and E) Depolymerizing of F-actin by treatment with LatA had no effect on the transverse ring of microtubules in the *GFP-TUA6* conical cell. Stage 8 flower bud of the *GFP-TUA6* transgenic line were immersed in a solution containing 0.5 μg/ml latrunculin A for 5-min treatment. To prevent repolymerization of the F-actin, the same treatment was repeated 24 h later for another two times. After treatments, stage 14 flower of the *GFP-TUA6* transgenic line was used for analysis of microtubule organization patterns. Scale bar = 2 μm. (F) Quantification of anisotropy of microtubule arrays showed that there was no significant difference between control and LatA treatment (One-way ANOVA, P = 0.478). Values are given as the mean ± SD of 10 cells.
(TIF)
