# Supplementary figures and images for: Spatio-temporal orientation of microtubules controls conical cell shape in Arabidopsis thaliana petals
Source: PLoS Genet. 2017 Jun 23;13(6):e1006851. doi: 10.1371/journal.pgen.1006851 (PMC5507347; doi:10.1371/journal.pgen.1006851)

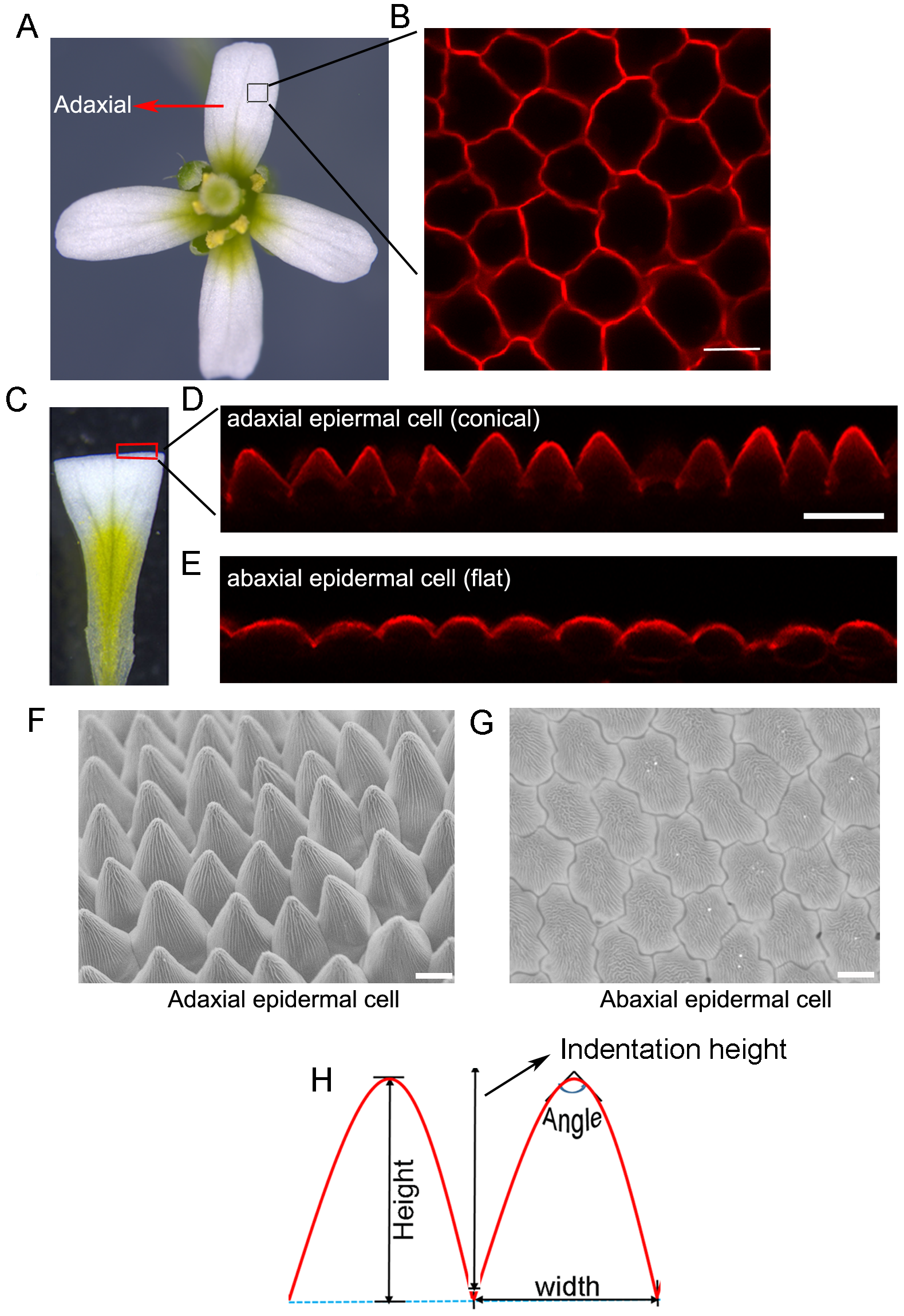

Supplement: S1 Fig — (A) A wild-type mature A. thaliana flower (stage 14) for observation of adaxial epidermal cell shape. The square area is visualized by confocal. (B) Confocal imaging analysis of petal adaxial epidermal cells from the top view of a petal sample. Scale bar = 10μm.(C) A folded back petal for observation of the serrated shape of conical cells from the side view of conical cells. The square area of the folded back petal allows for the side visualization of the conical cells by confocal. (D and E) Example confocal images of conical adaxial epidermal cells and flat abaxial epidermal cells from petals of Arabidopsis. Scale bars = 10μm. For the observation of conical adaxial epidermal cells and flat abaxial epidermal cells, adaxial epidermis and abaxial epidermis are folded back, respectively. (F and G) Representative images via a TM-3000 table-top scanning electron microscope view of A. thaliana petal adaxial epidermis and abaxial epidermis. The petal adaxial epidermis has conical-shaped cells (F), while the abaxial epidermis has flat-shaped cells (G). Scale bars = 10μm. (H) A cartoon depicting how cell heights, indentation heights, and cone angles are manually measured using the ImageJ software. (TIF) [file pgen.1006851.s001.tif]

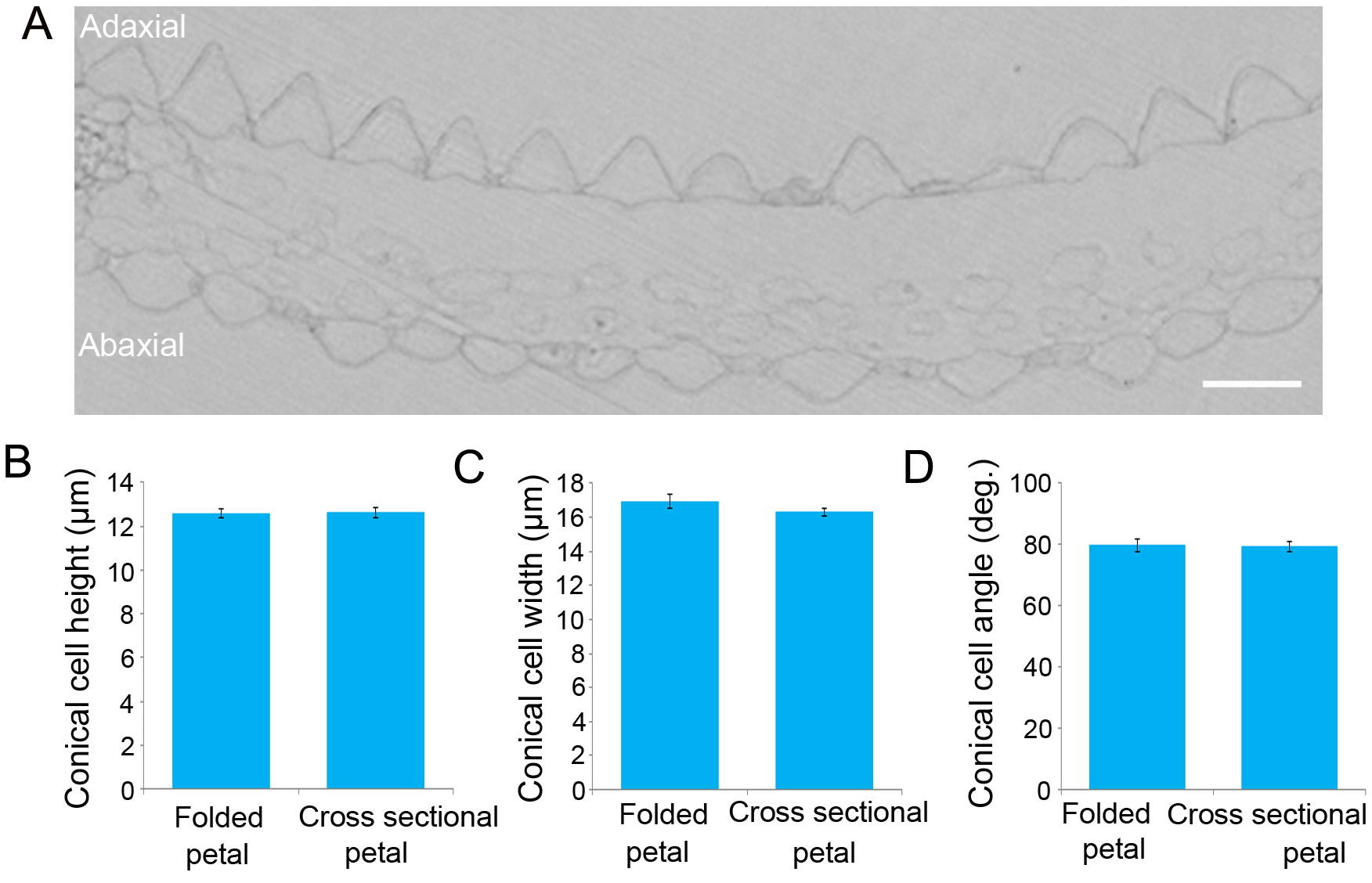

Supplement: S2 Fig — (A) A representative image of toluidine-blue stained cross section of a mature A. thaliana wild-type petal. Scale bar = 20μm. (B–D) Quantitative analyses of the geometry of wild-type conical cells. Propidium iodide-stained folded petals were visualized by confocal microscope, and toluidine-blue stained cross sectional petals were observed by optical microscope. Cell heights (B), cell widths (C), and cone angles (D) were quantified from the images made by these two imaging methods. Quantification data shows no significant differences of the geometry of conical cells from the images made by these two imaging methods [student’s t-test, P = 0.092 (B), P = 0.078 (C), and P = 0.124 (D)]. Values are given as the mean ± SD of more than 300 cells of 6 petals from independent plants. (TIF) [file pgen.1006851.s002.tif]

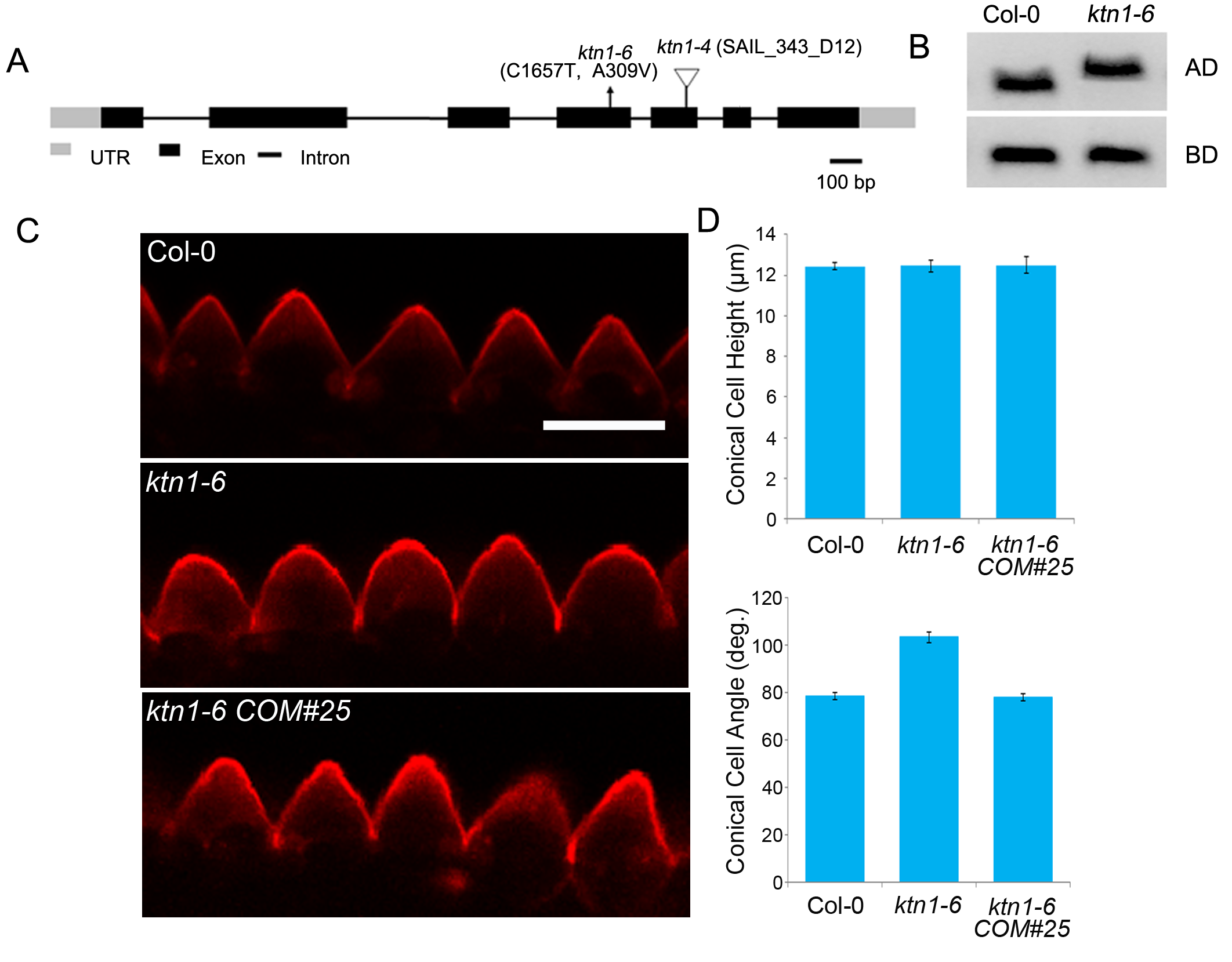

Supplement: S3 Fig — (A) The identification of the ktn1-6 and ktn1-4 mutants. (B) Identification of the ktn1-6 mutation by dCAPS1 marker. The ktn1-6 mutation disrupts the cleavage site of SpeI. (C and D) Complementation of the ktn1-6 mutant. Representative confocal images of the geometry of conical cells from wild type, ktn1-6, and the ktn1-6 complementation line (C). Complementation of ktn1-6 by transforming pKTN1::KTN1 into the ktn1-6 plants. More than ten complementation lines were obtained and one representative transgenic line(ktn1-6 COM#25) displaying similar conical cell shape to the wild type is shown. Scale bar = 10μm. Quantitative analyses of the geometry of conical cells (D). The average cell height and cell angle from the complementation line were similar to those of the wild type (student t-test, P = 0.672, P = 0.723). Values are given as the mean ± SD of more than 280 cells of 5 petals from independent plants. (TIF) [file pgen.1006851.s003.tif]

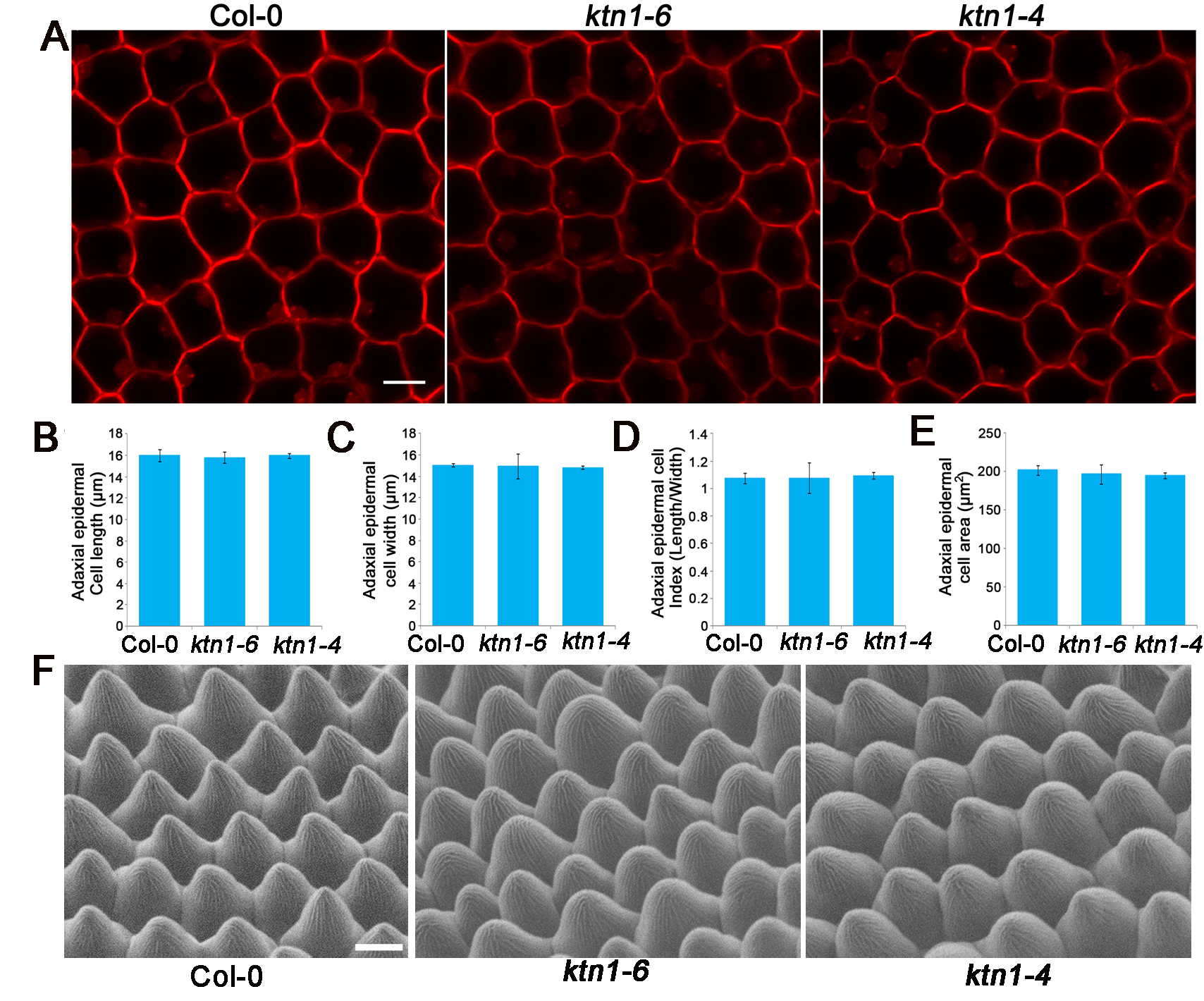

Supplement: S4 Fig — (A) Representative confocal images of the basal parts of conical cells. Mature petals at development stage 14 were used for imaging analysis of adaxial epidermal cells from the top view via confocal microscopy. The ktn mutants' cells showed similar hexagonal base to the wild type. Scale bar = 10μm. (B–E)Analyses of cell length (B), cell width (C), cell index (D), and cell area (E) showed that the hexagonal basal sizes of conical cells of the ktn mutants were similar to those of the wild type. Values are given as the mean ± SD of more than 200 cells of petals from independent plants. (F) Representative images via a TM-3000 table-top scanning electron microscope view of adaxial epidermis. The ktn1 mutants displayed increased isotropic apical expansion of conical cells compared with the wild type. Three independent experiments were conducted and showed similar results. Scale bar = 10μm. (TIF) [file pgen.1006851.s004.tif]

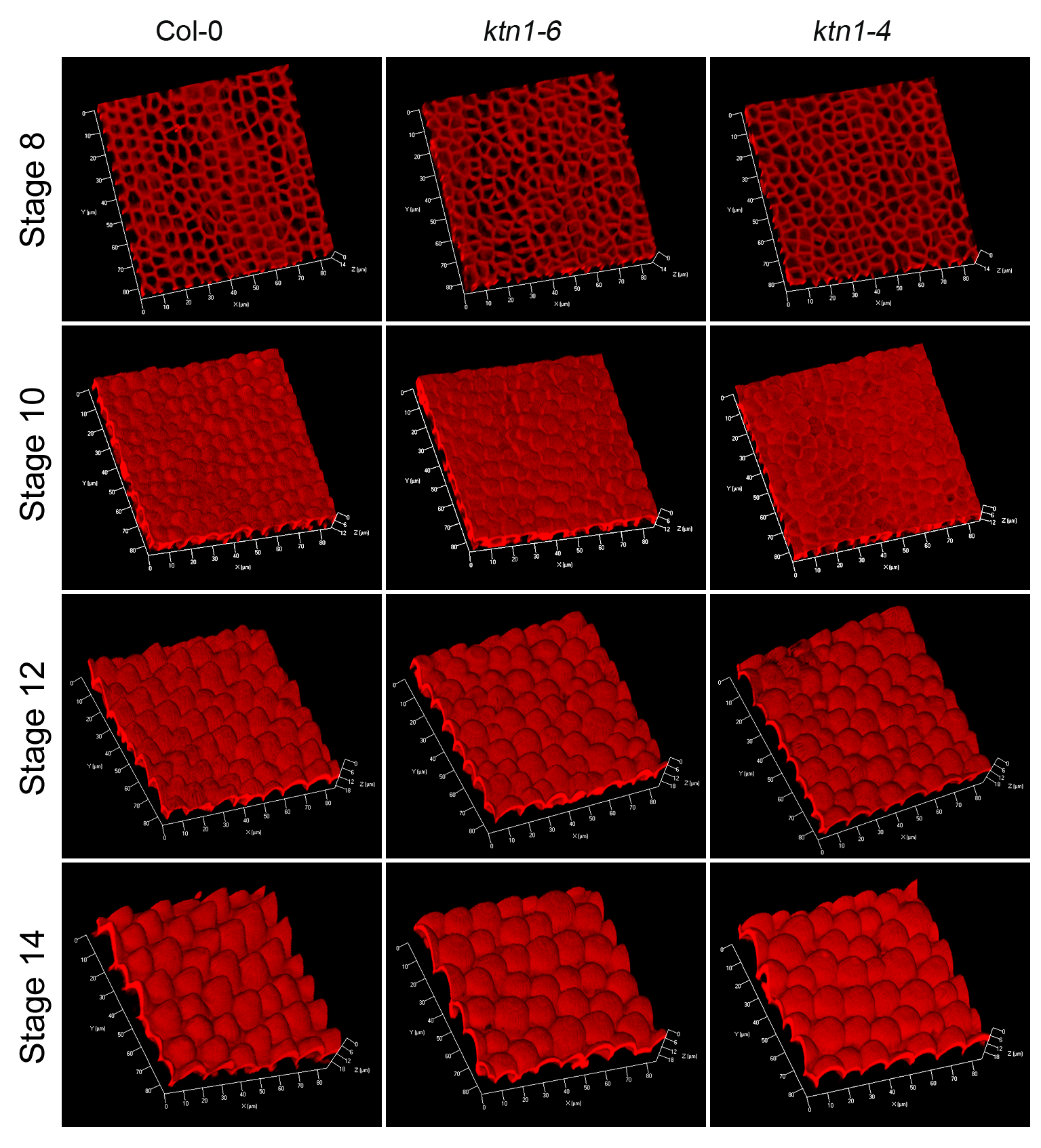

Supplement: S5 Fig — Representative images of 3D geometry of conical cells at the indicated developmental stages from wild type and the ktn mutants. Z stacks of confocal images from the distal regions of PI-stained petal samples from various developmental stages were taken from the top view along their Z axis at steps of 0.8 μm to reconstruct the 3D images. (TIF) [file pgen.1006851.s005.tif]

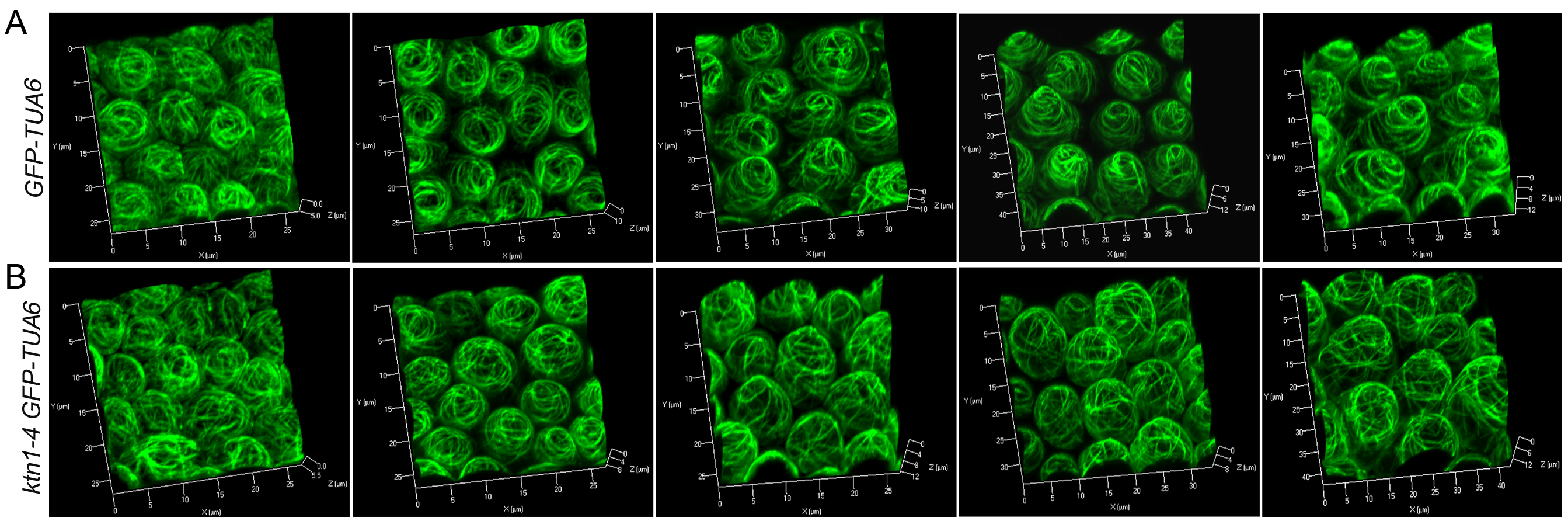

Supplement: S6 Fig — (A and B) 3D reconstructed microtubule configuration in wild-type (A) and the ktn1-4 mutant (B) conical cells stably expressing GFP-TUA6 at the indicated developmental stages. (TIF) [file pgen.1006851.s006.tif]

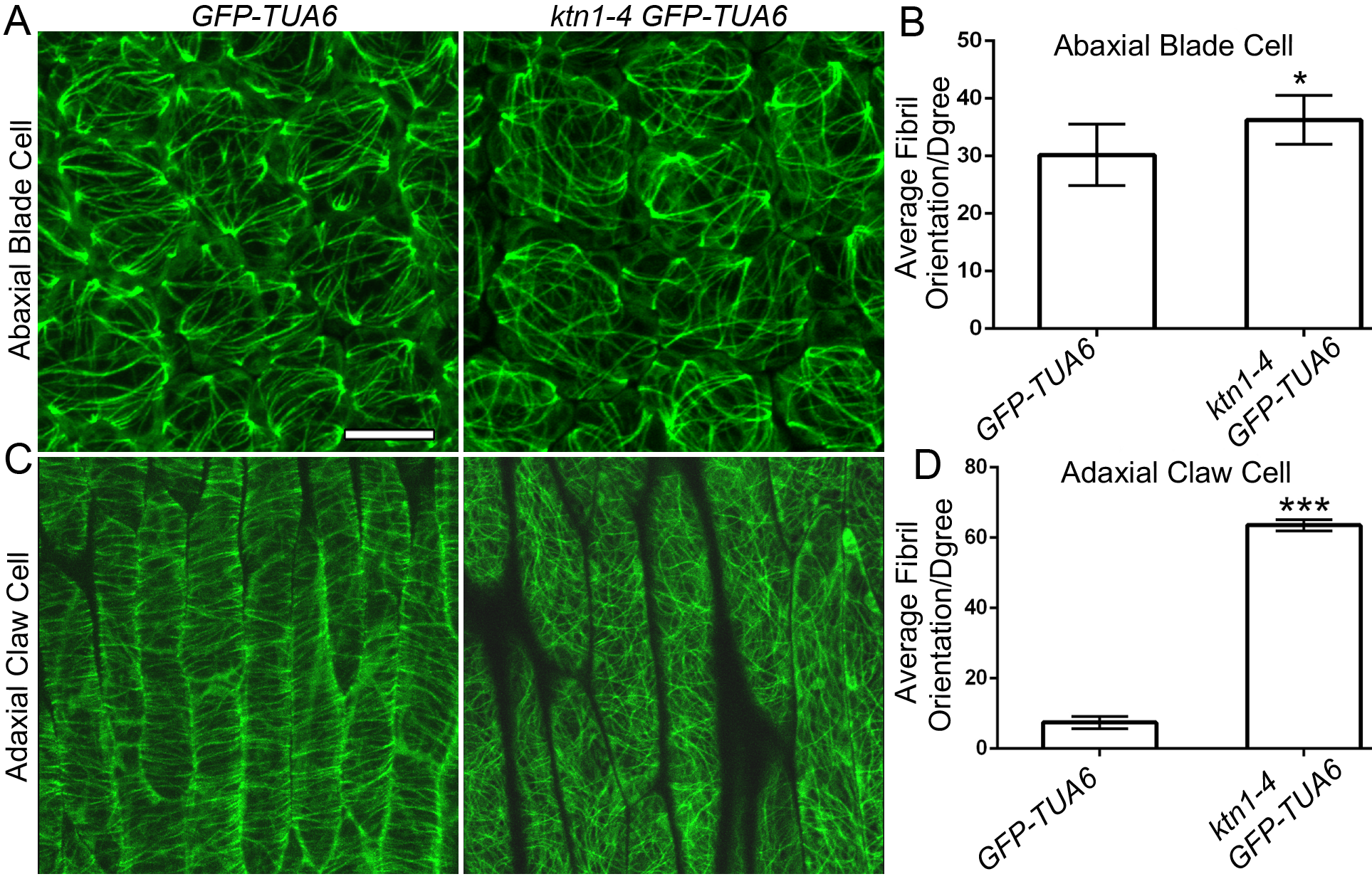

Supplement: S7 Fig — (A and C) Representative confocal images showing microtubule arrangement in petal abaxial blade epidermal cells (A) and adaxial petal claw cells (C) from both wild type and the ktn1-4 mutant stably expressing GFP-TUA6. Surface projections of confocal images from the abaxial epidermis of the non-folded petals. Scale bar = 10 μm. (B and D) Quantitative analysis of the average fibril orientation in abaxial blade epidermal cells (B) and adaxial petal claw cells (D) from wild-type and ktn1-4 petals. FribrilTool, an ImageJ plug-in, was used for quantification of the orientation angle. One-way ANOVA followed by Sidak's multiple comparison test indicated a significant difference (*P<0.05 and ***P<0.001) between the data sets from the GFP-TUA6 line compared with the ktn1-4 GFP-TUA6 line [P = 0.02302 (B), and P = 0.000000322 (D)]. Values are given as the mean ± SD of more than 100 cells of 3 petals from independent plants. (TIF) [file pgen.1006851.s007.tif]

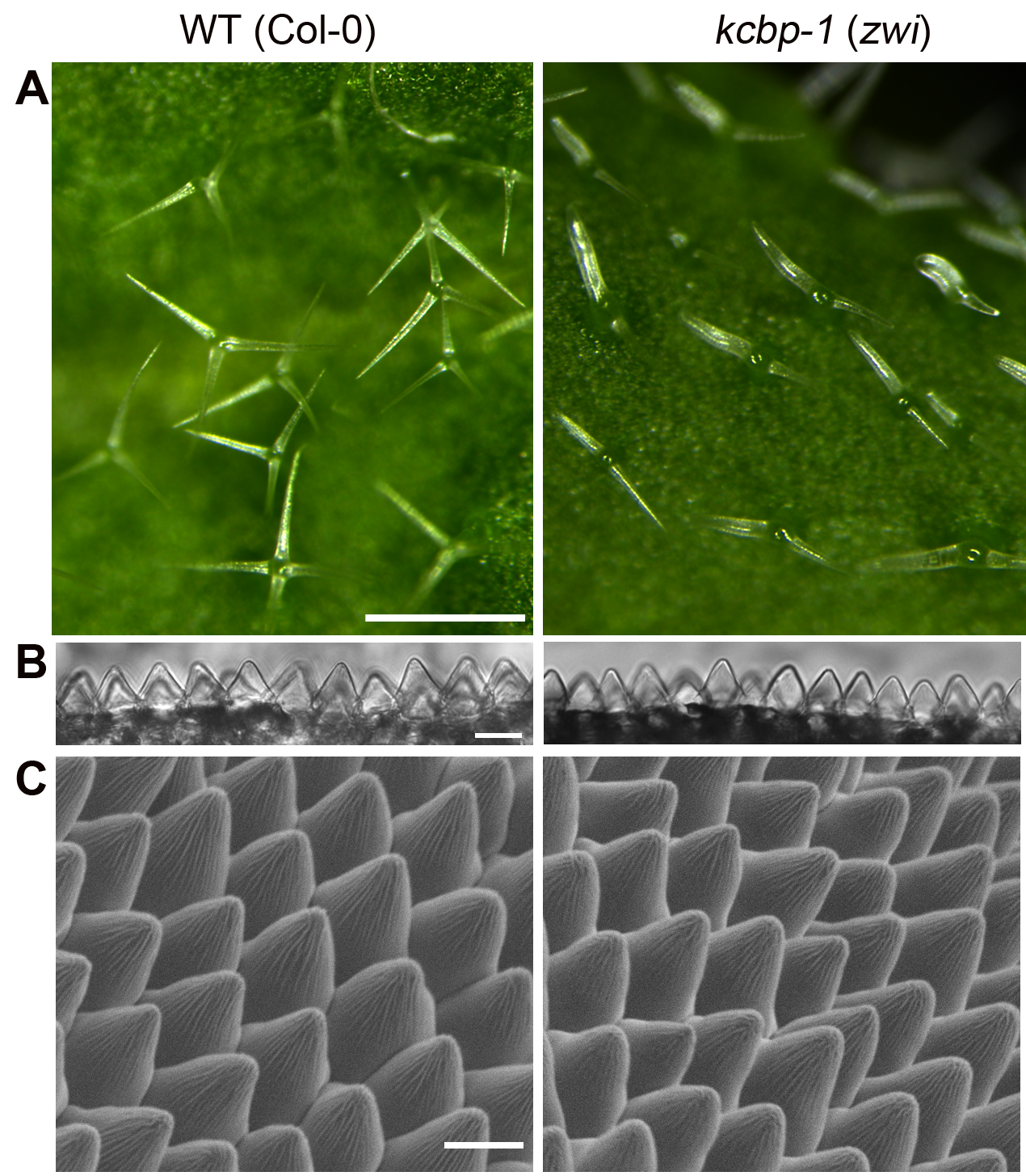

Supplement: S8 Fig — (A) Representative images via stereo microscope view of leaf trichomes in wild type and the kcbp-1 (zwi) mutant. Scale bar = 0.5 cm. (B) Example images of petal conical cells from wild type and the kcbp-1 mutant. Note that there is no obvious difference between wild type and the mutant. Scale bars = 10μm. (C)Representative images via a TM-3030 table-top scanning electron microscope view of conical cells from wild type and the kcbp-1 mutant. Note that there is no obvious difference between wild type and the mutant. Scale bars = 10μm. (TIF) [file pgen.1006851.s008.tif]

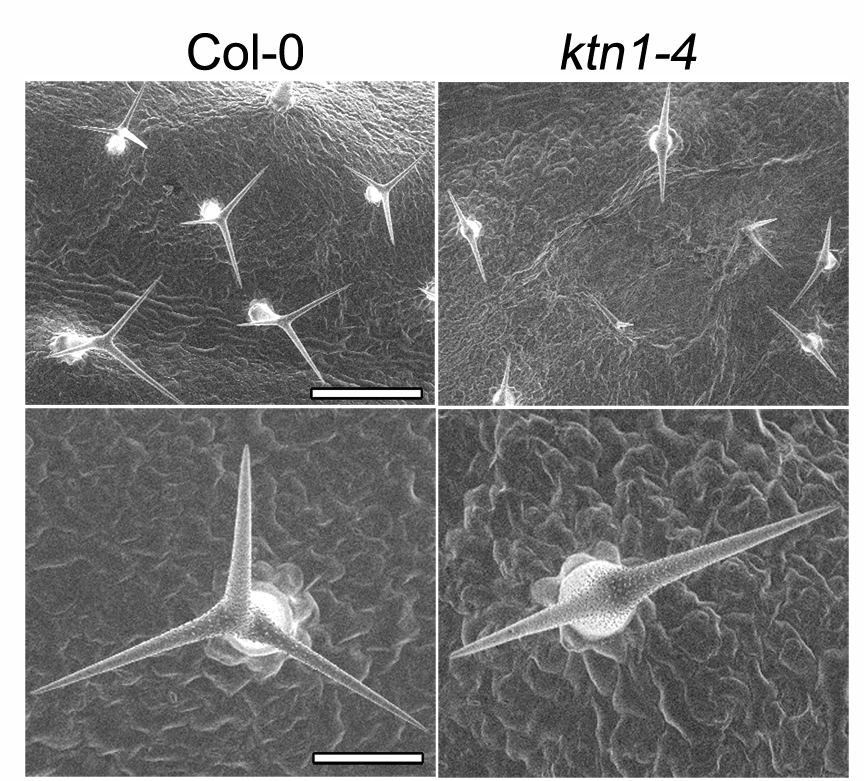

Supplement: S9 Fig — The ktn1-4 mutant has two-branch trichomes, displaying no swollen tips compared with the wild type. Scale bars in top and bottom panel represent 400 μm and 100 μm, respectively. (TIF) [file pgen.1006851.s009.tif]

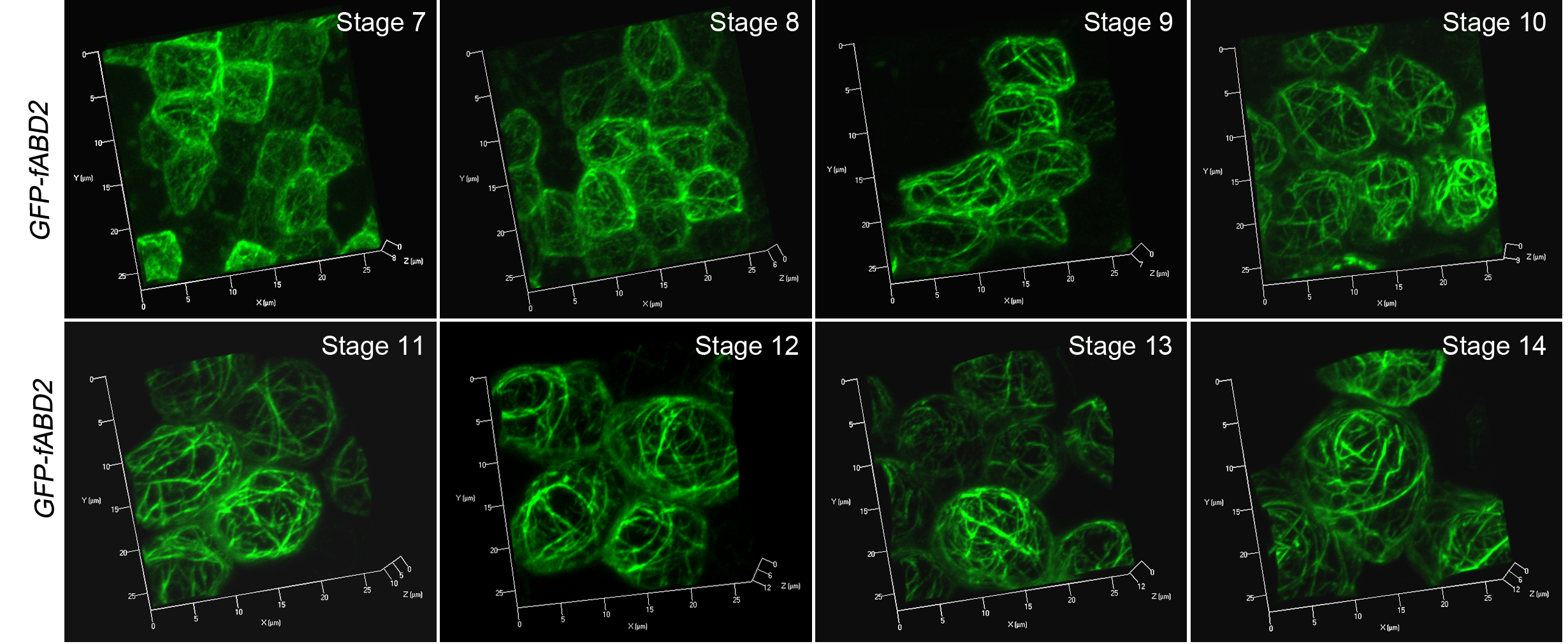

Supplement: S10 Fig — 3D reconstructed actin filaments configuration in wild-type the ktn1-4 conical cells stably expressing GFP-fABD2 at the indicated developmental stages. (TIF) [file pgen.1006851.s010.tif]

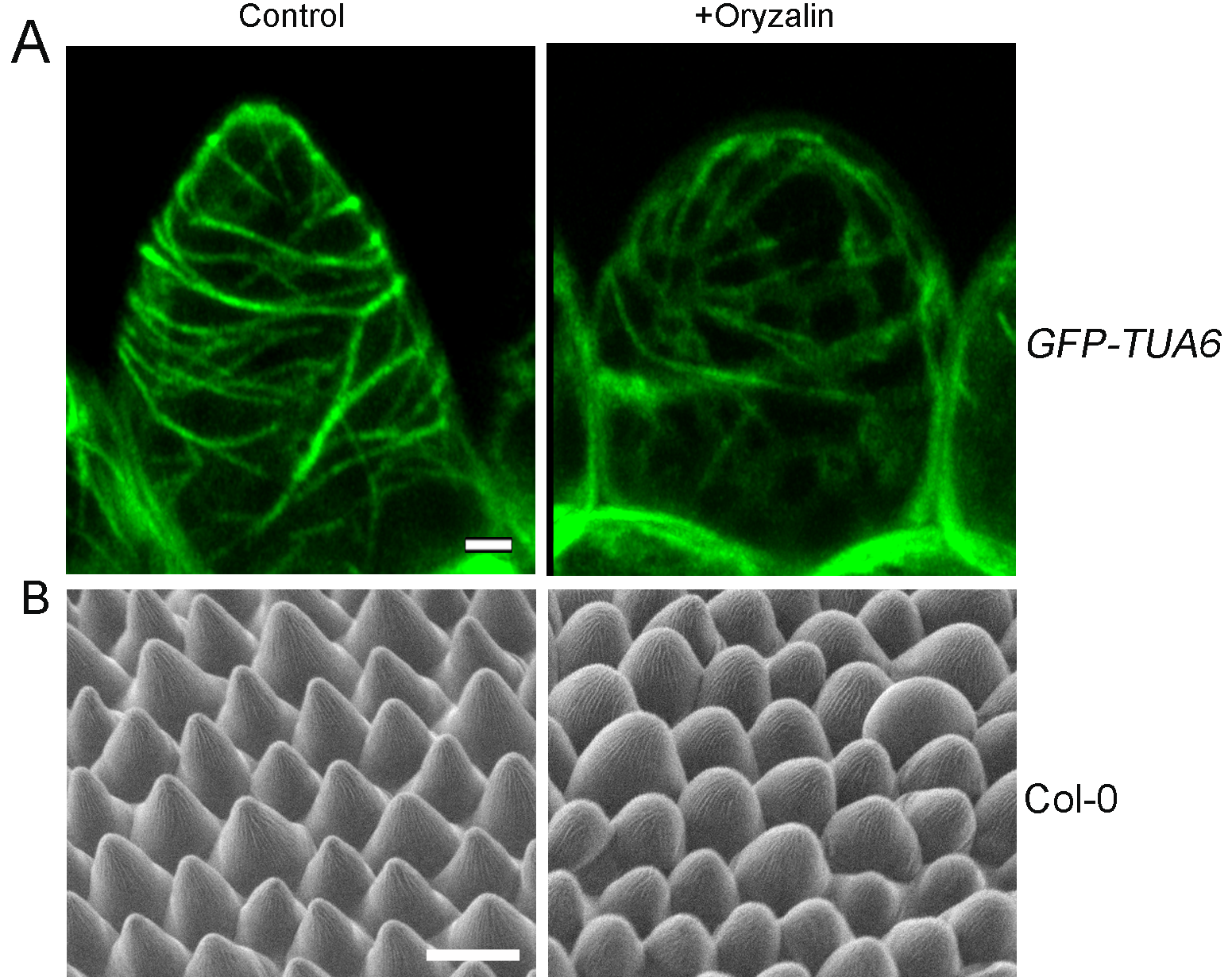

Supplement: S11 Fig — (A) Application of oryzalin showing depolymerization of microtubules. Stage 7 floral buds of the GFP-TUA6 transgenic line were immersed in a solution containing 30 μg/ml oryzalin for 5 min. To prevent repolymerization of the microtubules, the same treatment was repeated 24 h later for another two times. Microtubules in conical cells from petal development stage 14 were observed. Application of oryzalin could cause depolymerize microtubules in conical cells and increased isotropic cell expansion in the GFP-TUA6 transgenic line. Three independent experiments were conducted and showed similar results. Scale bar = 2 μm. (B) Representative images via a TM-3030 table-top scanning electron microscope (Hitachi) view of wild-type conical cells. Application of oryzalin caused increased isotropic cell expansion in conical epidermal cells. Three independent experiments were conducted and showed similar results. Scale bar = 10μm. (TIF) [file pgen.1006851.s011.tif]

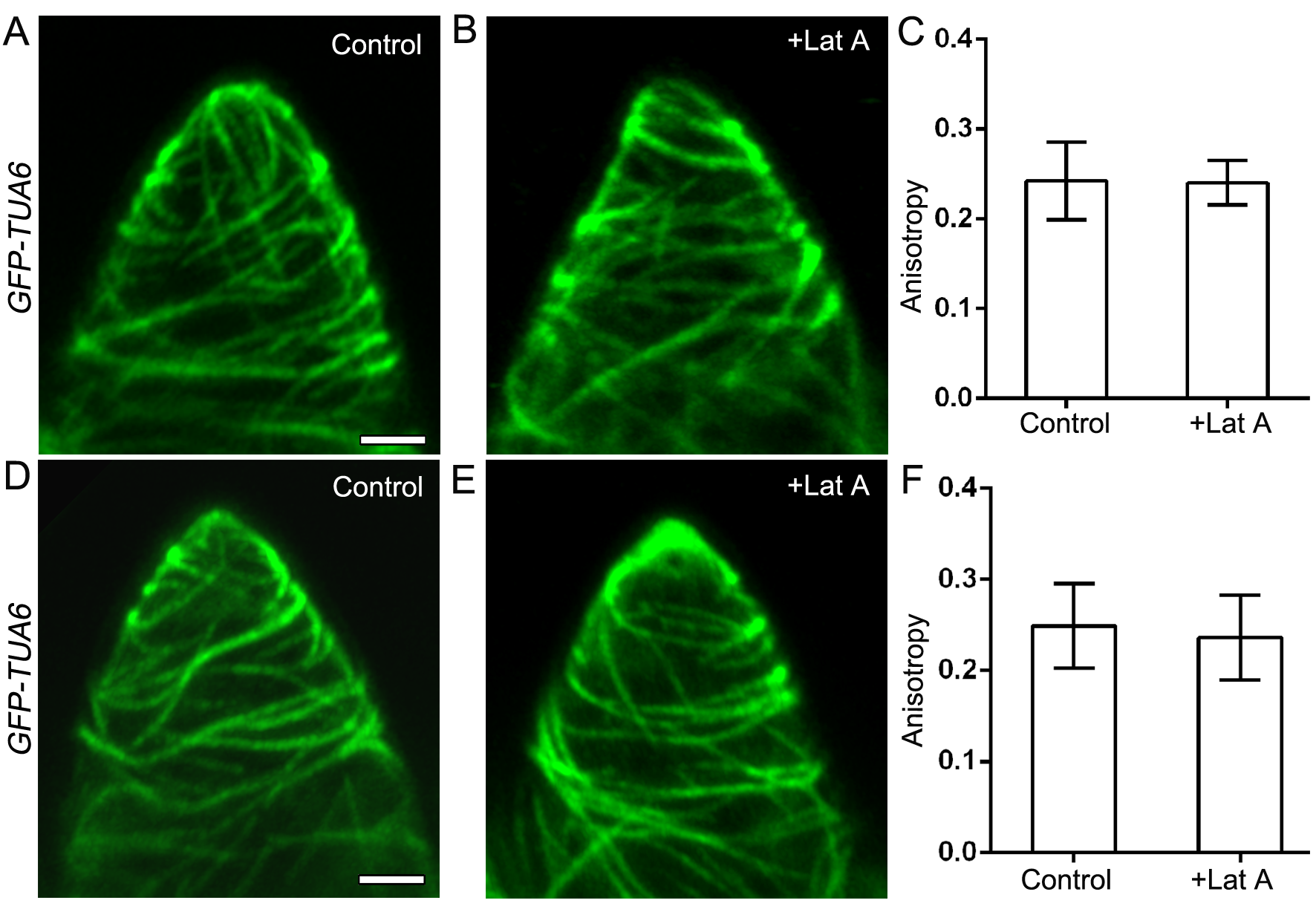

Supplement: S12 Fig — (A and B) Depolymerizing of F-actin by treatment with LatA had no effect on the configuration of transverse ring of microtubules in the GFP-TUA6 conical cell. Stage 14 flower of the GFP-TUA6 transgenic line were immersed in a solution containing 0.5 μg/ml latrunculin A for 15-min treatment. Scale bar = 2 μm. (C)Quantification of anisotropy of microtubule arrays showed that there was no significant difference between control and LatA treatment (One-way ANOVA, P = 0.652). Values are given as the mean ± SD of 10 cells. (D and E) Depolymerizing of F-actin by treatment with LatA had no effect on the transverse ring of microtubules in the GFP-TUA6 conical cell. Stage 8 flower bud of the GFP-TUA6 transgenic line were immersed in a solution containing 0.5 μg/ml latrunculin A for 5-min treatment. To prevent repolymerization of the F-actin, the same treatment was repeated 24 h later for another two times. After treatments, stage 14 flower of the GFP-TUA6 transgenic line was used for analysis of microtubule organization patterns. Scale bar = 2 μm. (F) Quantification of anisotropy of microtubule arrays showed that there was no significant difference between control and LatA treatment (One-way ANOVA, P = 0.478). Values are given as the mean ± SD of 10 cells. (TIF) [file pgen.1006851.s012.tif]
